# AMR-meta: a *k*-mer and metafeature approach to classify antimicrobial resistance from high-throughput short-read metagenomics data

**DOI:** 10.1093/gigascience/giac029

**Published:** 2022-05-18

**Authors:** Simone Marini, Marco Oliva, Ilya B Slizovskiy, Rishabh A Das, Noelle Robertson Noyes, Tamer Kahveci, Christina Boucher, Mattia Prosperi

**Affiliations:** Department of Computer and Information Science and Engineering, University of Florida, 2004 Mowry Road Gainesville, FL 32610, USA; Department of Computer and Information Science and Engineering, University of Florida, 432 Newell Dr, Gainesville, FL 32611, USA; Department of Veterinary Population Medicine, University of Minnesota, 1365 Gortner Avenue 225, St. Paul, MN 55108, USA; Department of Computer and Information Science and Engineering, University of Florida, 2004 Mowry Road Gainesville, FL 32610, USA; Department of Veterinary Population Medicine, University of Minnesota, 1365 Gortner Avenue 225, St. Paul, MN 55108, USA; Department of Computer and Information Science and Engineering, University of Florida, 432 Newell Dr, Gainesville, FL 32611, USA; Department of Computer and Information Science and Engineering, University of Florida, 432 Newell Dr, Gainesville, FL 32611, USA; Department of Computer and Information Science and Engineering, University of Florida, 2004 Mowry Road Gainesville, FL 32610, USA

**Keywords:** functional metagenomics, short reads, antimicrobial resistance, machine learning, matrix factorization

## Abstract

**Background:**

Antimicrobial resistance (AMR) is a global health concern. High-throughput metagenomic sequencing of microbial samples enables profiling of AMR genes through comparison with curated AMR databases. However, the performance of current methods is often hampered by database incompleteness and the presence of homology/homoplasy with other non-AMR genes in sequenced samples.

**Results:**

We present AMR-meta, a database-free and alignment-free approach, based on *k*-mers, which combines algebraic matrix factorization into metafeatures with regularized regression. Metafeatures capture multi-level gene diversity across the main antibiotic classes. AMR-meta takes in reads from metagenomic shotgun sequencing and outputs predictions about whether those reads contribute to resistance against specific classes of antibiotics. In addition, AMR-meta uses an augmented training strategy that joins an AMR gene database with non-AMR genes (used as negative examples). We compare AMR-meta with AMRPlusPlus, DeepARG, and Meta-MARC, further testing their ensemble via a voting system. In cross-validation, AMR-meta has a median f-score of 0.7 (interquartile range, 0.2–0.9). On semi-synthetic metagenomic data—external test—on average AMR-meta yields a 1.3-fold hit rate increase over existing methods. In terms of run-time, AMR-meta is 3 times faster than DeepARG, 30 times faster than Meta-MARC, and as fast as AMRPlusPlus. Finally, we note that differences in AMR ontologies and observed variance of all tools in classification outputs call for further development on standardization of benchmarking data and protocols.

**Conclusions:**

AMR-meta is a fast, accurate classifier that exploits non-AMR negative sets to improve sensitivity and specificity. The differences in AMR ontologies and the high variance of all tools in classification outputs call for the deployment of standard benchmarking data and protocols, to fairly compare AMR prediction tools.

## Introduction

Antimicrobial resistance (AMR) is the ability of microorganisms to resist the effect of drugs targeted to eliminate them [1] and is globally recognized as a threat to public health because it makes treatment of microbial infections harder, increasing the risk of disease spread and severity [2]. Data from 890 US hospitals collected on specific combinations of antibiotics and bacteria show that AMR caused an estimated 622,390 infections in 2017 [3]. Treating infections caused by AMR is clinically challenging because it requires identifying which drugs the infecting strain is susceptible to and then making a timely decision on the therapy to use. Notably, AMR is not limited to health care, as it represents a significant challenge also in animal and plant health, and thus in the entire ecosystem [4]. Therefore, detecting AMR in clinical, veterinarian, and botanical isolates is pivotal to curb the spread of AMR pathogens and reduce its impact. Although culture-based methods can accurately detect AMR, they are resource intensive with respect to trained personnel, monetary cost, and time [5]. Moreover, because only a fraction of bacterial species are cultivable with standard methods, culture-based methods are only applicable to a small number of bacteria. For these reasons, whole-genome and metagenomics sequencing has become an increasingly prevalent method for AMR characterization. The challenge that then arises is how to accurately identify and quantify the AMR genes from such sequencing data. To accomplish this, a number of different methods have been proposed. Despite the concordance between*in silico* genotypic and *in vitro* phenotypic resistance assessment, the uptake of AMR prediction tools for routine health care has been slow, and they showed discordant performance in clinical settings [6].

AMR prediction methods for metagenomics rely on comparison to databases of AMR genes. Two comprehensive and widely used AMR databases are the Comprehensive Antibiotic Resistance Database (CARD) [7, 8] and MEGARes [9,10]. CARD is thoroughly maintained, with monthly updates on AMR determinants that have (i) an associated peer-reviewed scientific publication, (ii) a DNA sequence available in GenBank, and (iii) clear experimental evidence of elevated minimum inhibitory concentration over controls. Currently, CARD integrates >3,000 reference sequences of AMR genes and >1,500 single-nucleotide polymorphisms, knowledge on resistance mechanisms, and specific antibiotic classes. CARD uses a manually curated process and ontology, named the Antibiotic Resistance Ontology (ARO, github.com/arpcard/aro), which describes the molecular relations of antibiotic resistance (e.g., acquired resistance genes, drug targets, AMR mechanisms). MEGARes [9]—and its most recent 2.0 update [10]—is a hand-curated AMR database designed for high-throughput sequencing data processing. MEGARes includes CARD genes and variants but uses a different annotation structure. Specifically, it is a multi-level hierarchy (type, mechanism, class, group) in the form of a direct acyclic graph, ensuring that 2 higher level ranks are not linked to the same lower level rank. The MEGARes annotation graph is therefore an optimal structure for ecological profiling and construction of AMR classifiers because, for example, it cannot result in conflicting sequence classification. MEGARes 2.0 currently includes ∼8,000 genes. Major improvements from its first release consist in the inclusion of antibacterial biocide and metal resistance genes.

For AMR classification of metagenomic samples from high-throughput sequencing, 1 class of methods is based on the use of sequence read aligners. One widely used tool in this category is AMRPlusPlus [9], which aligns all reads to MEGARes using BWA [11] and then post-processes the alignment to identify the genes that have >80% coverage from the alignment, providing the associated AMR annotation in the output. AMRPlusPlus 2.0 [10] is an improved version of AMRPlusPlus that is designed to be faster for large-scale projects. AMRPlusPlus 2.0 provides a post-alignment classification through the ResistomeAnalyzer (quality measure for nucleotide coverage of a reference sequence for a given read) and the RarefactionAnalysis (assessment of sequencing depth) modules. It also incorporates prediction of AMR due to single-nucleotide polymorphisms in housekeeping genes, using a curated set that matches CARD. Of note, CARD also performs AMR prediction for housekeeping genes via the Resistant Gene Identifier (RGI), available as a web-service and a command-line application. Although alignment-based methods have high precision [12], they can only classify reads that align to known AMR genes. Given that existing AMR databases are incomplete, a large portion of novel AMR genes may go undetected.

Another class of methods for AMR characterization is alignment-free, using a variety of approaches including substring (*k*-mer) matching and machine learning. ResFinder [13] and KmerResistance [14] process metagenomic reads by first constructing the set of all unique *k*-length subsequences (called *k*-mer spectrum) from the dataset. ResFinder 4.0 compares the set of unique *k*-mers to detect AMR genes and AMR-related chromosomal gene mutations based on an reference database built on a collection of chromosomal point mutations in bacterial pathogens [15], resistance genes from the Antibiotic Resistance Genes Database (ARDB) [16], and other literature sources [17]. The user is required to input a specific bacterial species for which the resistance is searched. Eight bacterial species are available. KmerResistance, as ResFinder, compares the set of unique *k*-mers to an ad hoc gene AMR reference database derived from the literature [18,19]. Specifically, KmerResistance uses exact co-occurring *k*-mer matching between a query sequence and the database, with a “winner takes all” strategy, i.e., multiple *k*-mer occurrences on different genes are resolved by selecting the one with highest frequency. Next, a quality measure of a whole AMR gene match is defined as a probability function of coverage (i.e., fraction of the genome covered by ≥1 *k*-mer) and depth (i.e., average number of times the *k*-mers in the match). Similar to alignment-based methods, ResFinder and KmerResistance are also restricted to identifying genes that are found in a specified database and, therefore, have limited ability to detect putative AMR sequences. Another limitation of the *k*-mer–based approaches is their low flexibility with respect to sequencing errors [14], possibly increasing false-negative rates in sequence classification.

Other alignment-free methods use machine learning classifiers to identify putative and known AMR genes, such as Resfams [20] and Meta-MARC [12], both based on hierarchical hidden Markov models (HMMs). Resfams [20] preprocesses high-throughput sequences by assembling them and translating the resulting contigs into amino acid sequences. Meta-MARC can predict AMR for an input sequence (either a short read or a longer assembled contig), according to the resistance class, group, and mechanism hierarchy defined in the MEGARes hierarchical data structure. Specifically, Meta-MARC is an ensemble of HMMs, each trained on a group of genes from MEGARes. A classification is performed by aggregating predictions from the lowest level of the MEGARes annotation hierarchy towards the highest level. Meta-MARC achieves better sensitivity, specificity, fraction of classified high-throughput sequence data, and number of AMR classes identified when compared to alignment matches and Resfams. However, the performance of Meta-MARC with short-read data is worse than classifying assembled contigs.

DeepARG [21] is a hybrid machine learning and alignment-based approach that leverages convolutional deep learning networks. The alignment module first translates the input sequences to amino acids and using DIAMOND [22], and then aligns the translated sequences to a custom AMR database created by merging CARD, ARDB [16], and manually selected AMR sequences from the Universal Protein Resource (UNIPROT). The deep learning model then predicts the AMR class for all aligned reads. Because the machine learning step is subsequent to the alignment one, de facto DeepARG is hindered by the limitations of alignment-based AMR prediction algorithms.

For completeness, it is worth mentioning AMR gene identification methods that are not specifically designed for high-throughput short-read metagenomic data. These methods take as input 1 or a combination of the following: single genes, specific genome strains, genomic or proteomic variants, and/or protein primary, secondary, or tertiary structures. Similar to the methods described previously, these methods use alignment and/or machine learning paradigms [23–30]. These algorithms restrict the user to performing 1 or more supplementary pre-processing steps on metagenomics data, not included into the algorithm, such as sequence alignment or assembly, sequence translations into proteins, or protein structure prediction. Because of the required pre-processing, these methods defy the very advantages provided by the alignment-free design. For further reference, Hendriksen et al. [31] provide a comprehensive review.

While our work focuses on raw short-read AMR classification, we duly note that in the wider field of computational microbiomics, a variety of bioinformatics approaches exist and can be combined at different levels, from the characterization of species diversity in commensal and pathogenic host-ecological settings, to the identification of *novel* AMR genes or genetic elements relevant to AMR mechanisms and evolution. The *de novo* assembly methods can reconstruct complete AMR genes from short-read data, locate them within core genomes or mobile elements, and assemble new genes that could be associated with phenotypic resistance; e.g., the MegaHIT project [32] assembled the world’s largest collection of gut microbiome genes with functional characterization. Also, the *de novo* assembly methods can be used to pre-process raw short-read data for AMR classification [29]. Fast alignment methods can be used as well to quickly identify genetic signatures or point mutations responsible for AMR, e.g., in housekeeping genes, and map very large metagenomics samples to databases of interest, such as 16S rRNA gene collections [33].

In this article, we develop AMR-meta, a novel, alignment-free, AMR classification approach for high-throughput metagenomic data, based on *k*-mers and matrix factorization of *k*-mers. The matrix factorization produces a number of "metafeatures" able to capture multiple levels of gene diversity within broad AMR classes. Importantly, and differently from existing methods, AMR-meta uses an augmented training strategy that incorporates non-AMR genes as negative examples. We show that our approach is competitive with state-of-the-art tools (i.e., AMRPlusPlus 2.0, Meta-MARC, and DeepARG) in classification performance and execution speed. Notably, AMR-meta captures resistance mechanics complementary to those found by other tools, which instead are more correlated to each other.

## Methods

**Figure 1:**
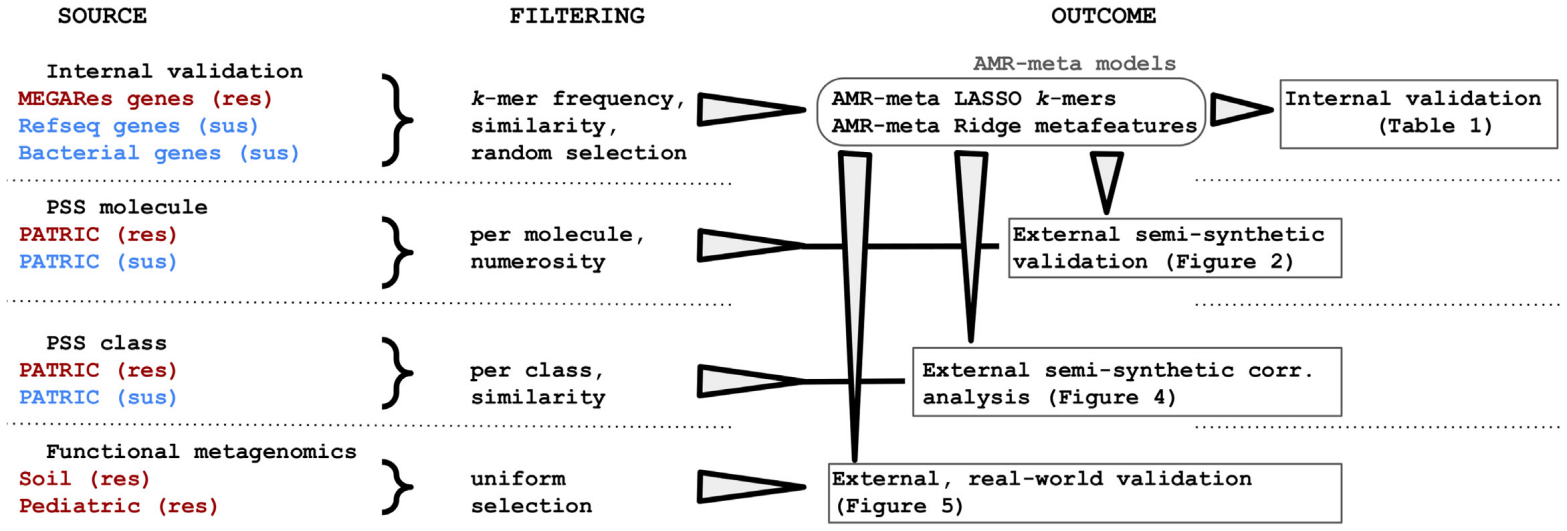
AMR-meta training/test workflow. We assemble an internal dataset of AMR and non-AMR homologous genes from MEGARes and RefSeq genes, on which AMR-meta models (*k*-mers, and metafeatures through matrix factorization) are trained and tested (70/30 split). AMR-meta and other AMR classification tools are then externally tested on (i) semi-synthetic data from PATRIC at both antibiotic class and molecule levels (PSS_cla_ and PSS_mol_) and (ii) functional metagenomics data (Soil and Pediatric).

AMR-meta is trained and tested first on an internal dataset that—differently from other approaches—includes both AMR (named "resistant") and non-AMR genes (named "susceptible"). The AMR genes are taken from MEGARes 2.0 [10], while non-AMR genes are chosen from Genbank’s RefSeq and include (i) bacterial genes that are highly dissimilar to AMR genes and (i) AMR-homologous sequences, i.e., sequences highly similar to AMR genes but not known to be associated with antibiotic resistance. By including the non-AMR and AMR-homologous sequences, we aim to decrease the false-positive calls and to increase the true-negative rates. This internal dataset is split into a 70/30 training/test ratio, and AMR-meta components (*k*-mers and *k*-mer–derived metafeatures) are trained and tested accordingly (all performance measures reported in this article are relative to test sets). Second, we generate 2 semi-synthetic external datasets, drawing bacterial genomes from the Pathosystems Resource Integration Center (PATRIC) [34], and simulating short-read data. We derive 2 PATRIC datasets that represent drug resistance/susceptibility relative to specific molecules or antibiotic classes, called PSS_mol_and PSS_cla_, respectively. This 2-fold design allows us to benchmark AMR-meta against other existing tools—AMRPlusPlus 2.0, Meta-MARC, and DeepARG—in a flexible way because their output levels vary among antibiotic classes and more specific mechanisms. We use PSS_mol_ to score the AMR predictions and PSS_cla_ to estimate the concordance of AMR-meta class predictions with those of other methods. Finally, we combine AMR-meta with the other tools and evaluate their predictions on 2 functional metagenomic datasets that were sampled from a clinical and an environmental setting. Our internal/external workflow is summarized in Fig. 1.

### Feature encoding and prediction models

#### AMR-meta *k*-mer LASSO module

The baseline models of AMR-meta are logistic regressors (1 for each antibiotic class) that use raw *k*-mers as input. Each model uses the whole class-specific *k*-mer spectrum (derived from the collated positive/negative training datasets), where each feature is a binary value, representing the presence or absence of a particular *k*-mer in the dataset. Given the high-dimensionality of the *k*-mer spectrum, we use least absolute shrinkage and selection operator (LASSO) regularization to reduce the feature space, optimizing the shrinkage operator via cross-validation [35]. Given the heterogeneity in gene diversity within each class (e.g., β-lactamases have higher diversity than fluoroquinolones), we also expect different cardinality of non-zero coefficients among the class-specific *k*-mer LASSO regressors.

#### AMR-meta metafeature ridge module

One possible problem with *k*-mer LASSO regression is that a single linear combination of *k*-mer features might not be able to explain the variance of the entire dataset, even if discrimination performance is good for the majority of genes in 1 class. A way to increase the portion of variance explained is to use >1 linear combination, e.g., the first *m*th vectors of a principal component analysis. In this way, multiple independent combinations of *k*-mers can more effectively represent the genetic diversity within antibiotic classes.

Accordingly, we explore a space transformation—with concomitant dimension reduction—of the *k*-mer spectrum that identifies a set of (orthogonal) multiple features, i.e., metafeatures, each as an independent combination of the original *k*-mers contributing to a cumulative portion of the data variance. To do so, we apply a matrix factorization approach, which has been previously shown apt to tackle complex feature extraction problems, e.g., oncology and proteomics [36,37]. The method is based on non-negative matrix tri-factorization [38]. The algorithm identifies low-rank, non-negative matrices whose product provides an approximation of the original non-negative matrix.

Here we consider 2 data domains, namely, *k*-mers and genes. A *k*-mer is related to a gene if it is present in the gene sequence. Let us denote the total number of genes with *g*; the total number of *k*-mers with *t*; a matrix of *r* rows and *c* columns having all values equal to zero with $\varnothing _{r,c}$; and a matrix with 1 gene per row and 1 *k*-mer per column with *R_g, t_*, and $R_{g,t}^T$ as its transpose. We denote the transpose of a matrix *A* with superscript *T* as *A*^*T*^ in the rest of this article. We express the relation between the 2 domains by a symmetrical, 4-block matrix:

\begin{eqnarray*}
R=\left({{\begin{array}{*{10}c}\varnothing _{g,g} & R_{g,t} \\ R_{g,t}^T & \varnothing _{t,t} \end{array}}}\right), \end{eqnarray*}

where non-diagonal block matrices represent the relation (intersections) between *k*-mers and genes. Note that in this context, the relation between elements is defined by design: We set the value of *R* at an entry to 1 if the corresponding *k*-mer is present in the corresponding gene, and 0 otherwise.

We denote the number of *k*-mer metafeatures and the number of gene metafeatures as *m_t_* and *m_g_*, respectively. The factorization procedure decomposes *R* into the product of 3 matrices *G, S*, and *G*^*T*^, such that *G* × *S* × *G*^*T*^ will approximate *R* by reducing the error up to a user-defined lower bound set as the difference between 2 consecutive iterations (denoted with *R* ≈ *GSG*^*T*^). Here *G* represents the relation between the original domains (genes, *k*-mers) and their metafeatures; and *S* represents the relation between the metafeatures, i.e., how 1 domain is mapped to the other. The matrices *G* and *S* have the following form, both expressed as 4-block matrices:

\begin{equation*} G=\left({{\begin{array}{*{10}c}G_{g,m_{g}} & \varnothing _{g,m_{t}} \\\varnothing _{k,m_{g}} & G_{t,m_{t}} \end{array}}}\right) and S=\left({{\begin{array}{*{10}c}S_{m_{g},m_{g}} & S_{m_{g},m_{t}} \\S_{m_{t},m_{g}} & S_{m_{t},m_{t}} \end{array}}}\right). \end{equation*}

We use the intersection between the data of the same domain as constraints in the factorization process; i.e., each domain has a block, symmetrical constraint. We define the matrix Θ to represent the self-domain relations, i.e., gene/gene and *k*-mer/*k*-mer relations. Therefore, Θ is an *R* × *R* matrix. The empty blocks of Θ are the non-diagonal blocks. $\Theta = \left ( _{\varnothing _{t,g}\,\,\,\Theta _{t,t}}^{\Theta _{g,g}\,\,\varnothing _{g,t}} \right )$.

In Θ we set each entry to −1 if the corresponding row and column elements share a relation; 1 if unrelated; and 0 if the relation is unknown. In this application, in the Θ_*t*_ block we consider each *k*-mer identical to itself (related, −1), while we make no assumption about the relation with 2 different *k*-mers (not related, 0). In the Θ_*g*_ block, we consider all the genes of each class to be related (−1), and different from the genes of other classes (1).

**Figure 2:**
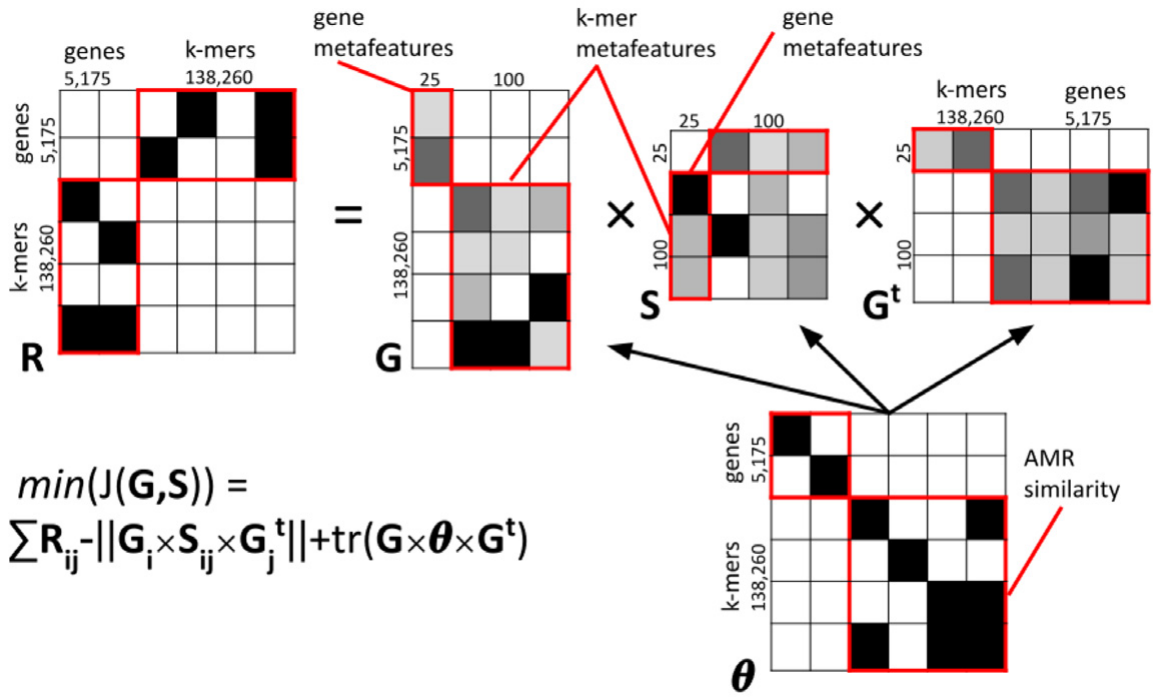
The matrix tri-factorization scheme. AMR, non-AMR, and AMR-homologous genes are paired up with *k*-mers across all antibiotic resistance classes into the *R* matrix, and the dimension is reduced through the *R* ≈ *GSG*^*t*^ factorization, where the metafeatures are extracted, revealing the AMR similarity phenotypes in the Θ matrix.

The goal of the factorization is to minimize the following objective function:

(1) \begin{equation*} \mathrm{ min}_{G \ge 0}(G;S) = \sum { ||R_{ij} - G_{i}S_{ij}G_{j}^{t} || + \mathrm{ tr}(G\Theta G^t)}, \end{equation*}

where || · || indicates the Frobenius norm and tr( · ) indicates the trace. The objective function is composed of 2 parts: The first part measures the difference between the original matrix and the product of the 3 factorized matrices; the second part calculates the adherence of the factorized metafeatures to the constraints, in our case based on the AMR resistance class. The factorization process proceeds in an iterative fashion until convergence to a local minimum, with convergence heuristically defined by observing the value of the objective function and the corresponding reconstruction error below a user-defined threshold [36–38]. We fix a threshold of 10^−2^ as the difference between consecutive iterations, or reaching 5,000 iterations, as stop criteria. Previous works discuss the method in detail [36,37]; a dedicated GitHub repository contains code and user manual [39]. The factorization process, calculated over the full-length training genes, produces $G_{{t},m_{t}}$, which is the matrix relating the *k*-mers to their metafeatures. For each short-read pair encoded as binary vector of *k*-mer occurrences sr_1, *t*_, we calculate its metafeatures as $\mathrm{ sr}_{1,t} \times G_{t,m_{t}}$. Because the optimal number of metafeatures can be hard to infer, and the sizes of the matrices grow with the number of features [36,37], for this application we used up to *m_t_* = 100 and *m_g_* = 25 metafeatures. After factorization, we feed the metafeatures to a logistic regression, optimizing the coefficients with a ridge approach. Figure 2 provides a graphical representation of the factorization process.

### Training strategy

#### AMR genes

We collate AMR genes from MEGARes 2.0 [10], constituting the positive (resistant) reference sets on the basis of the MEGARes annotation at the antibiotic class level. Of note, we exclude housekeeping genes that confer resistance through single point mutations.

#### Putative non-AMR bacterial genes

We include putative non-AMR genes from the RefSeq database [40]. Using BLAST, we select the 1,000 RefSeq bacterial genes that do not match to MEGARes (e-value = 10), aiming for a 1:1 target ratio with the antibiotic class of highest frequency. This gene set has high genetic divergence from the AMR genes in MEGARes, yet the nucleotide content is fully bacterial.

#### AMR-homologous human and vertebrate genes

To mimic genes that likely do not provide AMR but share a significant similarity with AMR genes, we assemble a dataset selecting AMR-homologous genes and gene fragments from the human genome (GRCh38), and all the contigs in RefSeq labeled as "vertebrate mammalian" and "vertebrate other" assemblies. To do so, we run an ungapped BLAST search of all MEGARes genes against these human and vertebrate sequences (e-value = 0.01). We use each unique sequence match, and add the flanking region to each match, elongating the matched sequence to be equal in length to the corresponding resistant MEGARes gene. Specifically, with a match of *n*_match_ nucleotides between target and query AMR gene, we extend the match by *n*_match_/2 nucleotides in both directions on the target MEGARes sequence. The underlying assumption here is that matches of bacterial AMR genes on vertebrate genomes are spurious or not functional and therefore do not provide AMR. Of note, this set-up is similar to the test set derivation presented in DeepARG [21].

#### k-mer–based and metafeature modeling

All *k*-mers present in the genes of the training datasets, excluding any sample reserved for validation (see next subsection), are considered and counted using different values of *k*, from 13 to 77 based on prior literature evidence [14]. The best value for *k* is chosen incrementally on the basis of internal validation performance, stopping when performance decreases. Next, we stratify the training samples by class. We remove all *k*-mers with a frequency less than a given cut-off *f* in a single class (3 or 5 upon internal validation). We also exclude AMR classes with <10 *k*-mers after frequency filtering.

#### Simulation of metagenomic short-read data for training

We use the AMR datasets described above to generate short reads, labeling each as resistant or susceptible to an antibiotic class. For each MEGARes class, we generate short-read datasets providing 10× base coverage of the original full-gene data. These datasets allow the evaluation of both false-positive and false-negative results.

### External validation

We use 4 independent external datasets, 2 semi-synthetic (made similarly to the training set) and 2 from functional metagenomic experiments. Because the prevalence of AMR and the *k*-mer spectrum in the external test set is not guaranteed to be balanced as in the training, we re-calibrate the *k*-mer and metafeature probability threshold for external validation using the internal validation dataset and a number of samples where the *k*-mer and metafeature vectors are empty; i.e., they represent the non-AMR gene background. The ratio is optimized between 1:0.05 and 1:10, picking the first that meets the calibration target, i.e., a prediction with a score <0.5 for a feature vector without any *k*-mer belonging to our model.

#### Semi-synthetic datasets

We create the semi-synthetic datasets from PATRIC, downloading via FTP full bacterial genomes and summary metadata [23,30]. We retain only genomes annotated as susceptible or resistant after an antibiogram test conforming to the Clinical & Laboratory Standards Institute (CLSI), which is the most frequent testing standard in PATRIC, with >55,000 resistant and 54,000 susceptible records [30]. Because the antibiotic nomenclature in PATRIC is molecule-specific and does not match the MEGARes ontology hierarchy exactly, we compile a lookup table linking each PATRIC drug annotation to a MEGARes class. We remove PATRIC genomes that do not refer to the AMR classes considered in the training phases or are not included in the classes predicted by the concurrent methods.

We then generate 2 PATRIC semi-synthetic datasets (PSS), based on PATRIC antibiotic molecule labels (PSS_mol_) and MEGARes classes (PSS_cla_), respectively.

We use PSS_mol_ to assess the performance of our approach and the concurrent methods on molecule-specific data. We retain genomes that are resistant (or susceptible) to ≥1 MEGARes class. We rank the PATRIC drug labels on the basis of number of associated genomes, and we select the top ones on the basis of the associated MEGARes classes. We exclude labels with <250 genomes or labels not referring to a specific molecule (e.g., tetracycline). We generate 250,000 short reads for each PATRIC label, equally divided between resistant and susceptible. Note that for PSS_mol_, because the PATRIC labels refer to genome (and not the specific gene, as in MEGARes), it is not possible to determine the ground truth, i.e., whether a short read comes from a resistant or a susceptible gene. To assess each method's performance, in the absence of such ground truth, we develop a scoring system based on the assumption that a method should find more resistant read pairs from resistant genomes and fewer from susceptible genomes. With sr_res, res_ defined as the number of short-read pairs coming from resistant genomes and classified as resistant, and with sr_res, sus_ as the number of short-read pairs coming from susceptible genomes and classified as resistant, we define the *S*-score as *S* = sr_res, res_ − sr_res, sus_. A higher *S*-score thus denotes better performance, and a negative value implies that the method finds more resistant short-read pairs among the susceptible ones.

PSS_cla_ is collated at the class level. Unlike PSS_mol_, each short read from PSS_cla_ has a known label that indicates whether it comes from a resistant or susceptible gene. To generate PSS_cla_, first we remove PATRIC genomes presenting inconsistent class annotations, i.e., that are annotated as both resistant and susceptible to antibiotics belonging to the same class. Second, to consider only genomes that are resistant (or susceptible) to the range of antibiotics within a given MEGARes class, we rank each genome in decreasing order of the total number of annotations of resistance (or susceptibility) to multiple drugs within the same class. On the basis of this ranking, we retain only genomes that rank above the 90th percentile. Third, we perform a class-by-class BLAST filtering (e-value = 0.01, percent identity ∈ [70, 90]) of the selected PATRIC genomes against MEGARes genes, retaining and clipping the unique genes of PATRIC genomes that match MEGARes. The objective is to extract a set of PATRIC genes that match to MEGARes genes but are not exact matches. In fact, genes similar to known resistant genes coming from antibiotic susceptible—by a phenotypic test—genomes represent excellent candidates to test the ability of a classifier to recognize true/false-positive results. From these selected PATRIC genes, we generate short reads covering the selected genes, and capping the number of resistant or susceptible paired reads at ≤100,000 per AMR class (i.e., 400,000 total reads per class). We reckon that with this procedure, we are able to uniquely label each PATRIC instance that passes the filter; however, in the BLAST alignment, there could be flanking regions or inserts that produce artifact matches. Nonetheless, given the strict parameters used, we we deem these cases to be rare. A resistant sample likely contains only resistant reads, and vice versa for a susceptible sample. Therefore, it is possible to calculate sample-wide performance by counting the proportion of resistant-within-resistant and susceptible-within-susceptible reads in each test sample. After filtering, glycopeptides and lipopeptides are excluded because there are <15 resistant genomes. Sulfonamides are excluded because no susceptible genomes are retained by our filtering procedure.

#### Functional metagenomics data

We benchmarked our method against 2 functional metagenomic datasets, which we refer to as the Pediatric and the Soil datasets (NCBI BioProject Accessions PRJNA244044 and PRJNA215106, respectively). A functional metagenomics experiments is made by cloning metagenomic DNA fragments into bacterial vectors grown on antibiotic-laden media. The cultured bacteria surviving the antibiotic exposure are sequenced using a clonally amplified high-throughput sequence library. As per experimental design, for each fosmid, all sequence reads contain ≥1 AMR gene (known or not yet discovered) resistant to a known antibiotic. Therefore, each sequencing experiment has a known antibiotic resistance label. However, because the original metagenomics fragments can be longer than a single AMR gene, a single fosmid might contain multiple AMR genes, or contain unknown genes. The Pediatric and Soil datasets include fosmids from *Escherichia coli*(DH10B) and consist of of 219 and 169 samples with an average of 1.98 and 1.12 million paired-end short reads, respectively, sequenced with Illumina Genome Analyzer IIx technology. We use the aforementioned PATRIC annotation look-up table to pair antibiotic annotations to MEGARes classes. For testing classifier performance, we randomly select 100,000 short-read pairs for each class as for the PATRIC datasets.

### Software and hardware set-up

We process the training/validation data, the semi-synthetic PSS_mol_ and PSS_cla_ datasets, and the experimental functional metagenomics data through in-house UNIX scripts, off-the-shelf bioinformatics tools including BLAST, R, and Bioconductor. The *k*-mer LASSO and the metafeature regression are developed in R, bash, and C++. We download the functional metagenomics datasets using NCBI’s sra-toolkit. For short read generation, we use InSilicoSeq [41], simulating Illumina’s NovaSeq (thet company’s top-line production-scale sequencing instrument) reads with default parameters. We exclude genes shorter than 151 bases (length of NovaSeq’s short reads) from the simulations. Code and R scripts are available publicly at the project home page.

## Results

### AMR-meta provides competitive prediction performance on multiple AMR classes

We generate 13 datasets, corresponding to the following antibiotic classes (according to the MEGARes ontology): aminoglycosides, β-lactamases, drug and biocide resistance, fluoroquinolones, glycopeptides, lipopeptides, macrolide-lincosamide-streptogramin (MLS), multi-biocide resistance, multi-drug resistance, multi-metal resistance, phenicols, sulfonamides, and tetracyclines. We exclude classes with <10 *k*-mers after frequency filtering. Upon internal validation, the best *k*-mer length *k* and frequency threshold *f* are 13 and 5, respectively (the performance decreases at *k* = 31 and for *f*= 3 with the same or higher *k*). Upon optimization of the *k*-value, the total number of unique 13-mers is 138,260, and the median number per class is 3,645 (interquartile range [IQR], 1,658–7,168). The matrix factorization includes 5,175 training genes, yielding a matrix *R* of 138,260 + 5,175 = 143,435 rows and columns, and a *k*-mer/metafeature matrix of 138, 260 × 100 elements.

Table 1 shows the class-specific performance summaries by *k*-mer and metafeature regression on the internal validation sets. On the internal validation set, the *k*-mer LASSO and the metafeature regression exhibit a good trade-off between sensitivity and specificity at both *k*-values. The median number of features selected by *k*-mer LASSO is 12,783 (IQR, 12,304–13,179). As expected, the highest number of non-zero coefficients is found in the β-lactamase class, which is the class with higher diversity and number of resistant genes in MEGARes. The same holds for the highest number of metafeatures with positive coefficients (note that each metafeature is derived from the matrix factorization described above, incorporating several hundreds of thousands of *k*-mer/gene elements). In terms of performance, for LASSO, the median (IQR) f-measure across all classes is 0.7 (0.2–0.9), while for the metafeature regression, the median f-measure is 0.4 (0.2–0.7). For both methods, the best-performing classes are β-lactamases and fluoroquinolones, while the most problematic are MLS, and multi-biocide, -drug, and -metal resistance. Despite the *k*-mer LASSO having a higher median f-measure, the metafeature regression performs better in the problematic MLS and drug and biocide classes and shows better sensitivity in glycopeptides and better specificity in fluoroquinolones and lipopetides. For reference comparison, the median f-measure across classes is 0.5 (IQR, 0.3–0.7) for DeepARG and 0.9 (IQR, 0.9–1.0) for Meta-MARC, based on the original articles’ validation results. AMRPlusPlus 2.0 does not report per-class results on test sets.

**Table 1:** Performance of *k*-mer LASSO and metafeature ridge regression in predicting antibiotic class susceptibility/resistance on the internal test sets (30% of full dataset)

		*k*-mer LASSO					Metafeature ridge				
Antibiotic class	N (test)	No. features	f-measure	MCC	Sensitivity	Specificity	No. metafeatures	f-measure	MCC	Sensitivity	Specificity
Aminoglycosides	4,920	13,162	**0.85**	**0.84**	**0.79**	**0.99**	54	0.58	0.54	0.57	0.97
β-lactamases	36,052	19,483	**0.96**	**0.93**	**0.94**	**0.99**	74	0.89	0.79	0.83	0.96
Drug and biocide resistance	5,055	13,064	0.36	0.39	**0.93**	**0.76**	56	**0.39**	**0.93**	0.7	0.66
Fluoroquinolones	1,286	11,462	**0.98**	**0.98**	**0.96**	**1**	50	0.9	0.9	0.92	**1**
Glycopeptides	3,200	12,700	**0.8**	**0.8**	0.7	**1**	54	0.23	0.27	**0.84**	0.75
Lipopeptides	1,084	12,356	**0.85**	**0.85**	**0.76**	**1**	43	0.8	0.8	0.73	**1**
Macrolide-lincosamide-streptogramin	2,210	14,064	0.2	0.28	**0.93**	0.77	54	**0.3**	**0.29**	0.38	**0.97**
Multi-biocide resistance	1,412	12,304	**0.13**	**0.2**	**0.88**	**0.76**	51	0.1	0.16	0.78	0.73
Multi-drug resistance	1,387	12,280	**0.13**	**0.21**	**0.91**	**0.77**	48	0.11	0.18	0.83	0.74
Multi-metal resistance	2,407	13,179	**0.21**	**0.28**	**0.92**	**0.76**	62	0.18	0.25	0.9	0.73
Phenicols	922	11,115	**0.74**	**0.74**	**0.66**	**1**	51	0.44	0.44	0.53	0.99
Sulfonamides	531	12,783	**0.75**	**0.78**	0.6	1	54	**0.75**	0.77	1	0.6
Tetracyclines	4,208	14,286	0.86	0.85	0.8	1	43	0.67	0.65	0.67	0.98

Results show f-measure, Matthews correlation coefficient (MCC), sensitivity, and specificity; also, the number of non-zero *k*-mer LASSO and positive metafeature ridge coefficients are shown.

### AMR-meta generalizes robustly on external, semi-synthetic datasets

**Figure 3:**
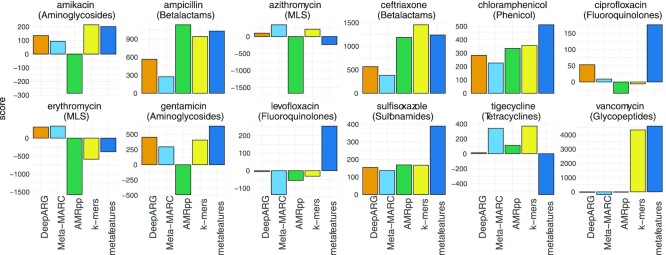
Performance of AMR-meta (*k*-mers and metafeatures) and of other off-the-shelf tools on the molecule-level PATRIC semi-synthetic data (PSS_mol_). The *S*-score is the difference between short-read pairs predicted as resistant from the pooled resistant and susceptible genomes drawn from PATRIC.

The PSS_mol_ dataset includes 12 molecule labels incorporated into antibiotic classes, namely, ciprofloxacin and levofloxacin (fluoroquinolones), gentamicin and amikacin (aminoglycosides), ceftriaxone and ampicillin (β-lactamases), chloramphenicol (phenicols), sulfisoxazole (sulfonamides), erythromycin and azithromycin (MLS), tigecycline (tetracyclines), and vancomycin (glycopeptides). Performance results in terms of *S*-score, which summarizes the correct resistance and susceptible hits (the higher the better), are shown in Fig. 3. The median *S*-score for the *k*-mer LASSO is 285.5 (IQR, 123.5–540), and for the metafeature regression is 322 (IQR, 73–470). Meta-MARC scores 250 (IQR, 72–359.5), DeepARG scores 144.5 (IQR, 43–345), and AMRPlusPlus 2.0 scores −29 (IQR, −377.5 to 210). Overall, our metafeature approach shows both the highest performance and stability, also exhibiting a positive score in the levofloxacine molecule, whereas all the other methods produce a negative score. The *k*-mer LASSO component ranks second, followed by the other off-the-shelf tools.

### AMR-meta predictions complement those of existing algorithms

**Figure 4:**
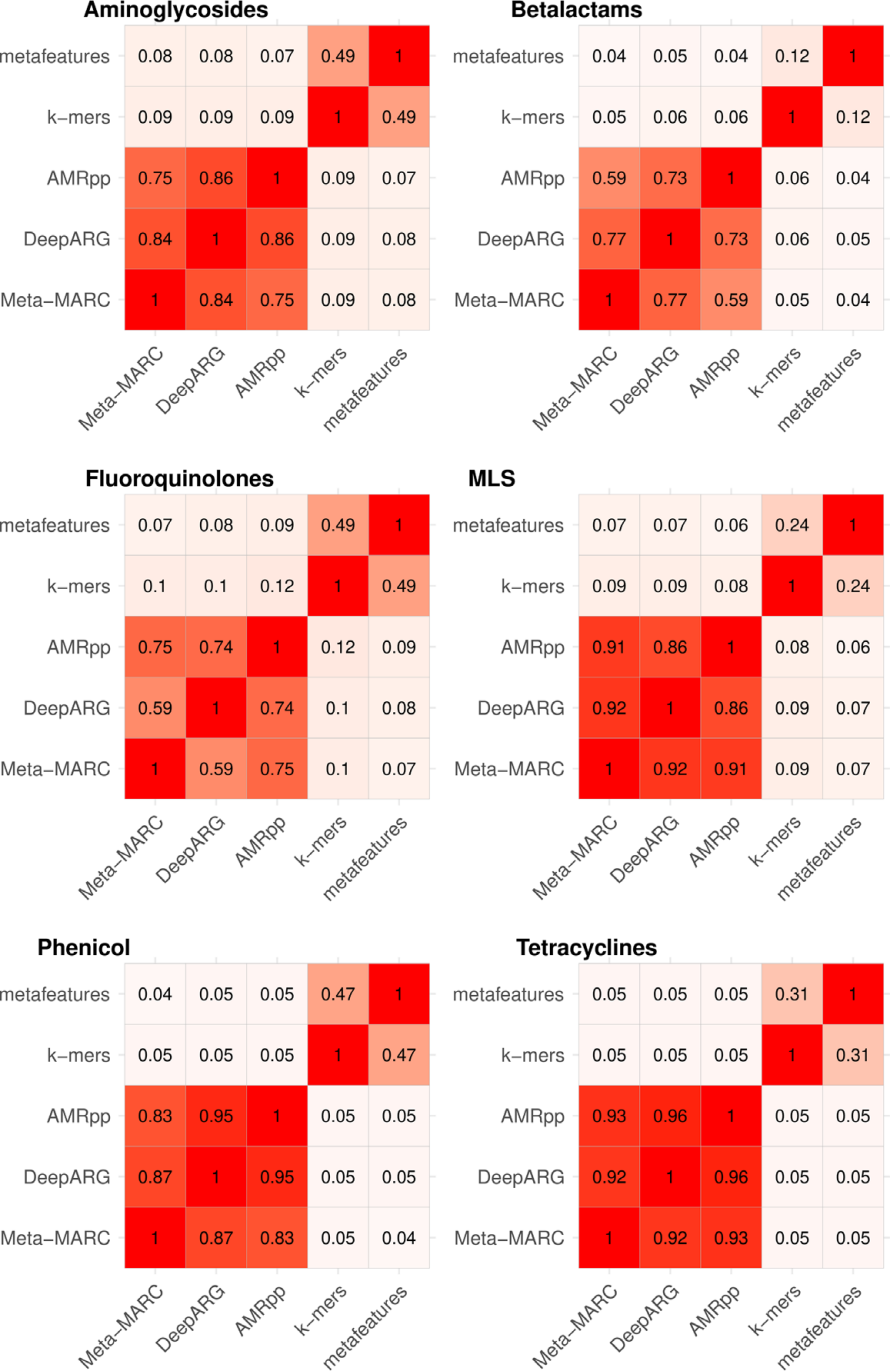
Spearman rank correlation of the AMR classifiers on the PATRIC semi-synthetic data (PSS_cla_).

Next, we measure the correlation between the predictions of the 2 AMR-meta modules and the ones from the other algorithms. Because PSS_mol_ does not have a per-gene defined ground truth, we assemble PSS_cla_. The PSS_cla_ dataset includes 6 of the 13 MEGARes classes, namely, aminoglycosides, β-lactamases, fluoroquinolones, MLS, phenicols, and tetracyclines. PSS_cla_ has instances from both positive (resistant) and negative (susceptible) genes. When we look at the class-specific concordance for each pair of tools using the the Spearman rank correlation (Fig. 4), PSS_cla_ shows that the algorithms behave differently. Specifically, DeepARG, Meta-MARC, and AMRPlusPlus 2.0 are highly correlated in most of the antibiotic classes (range 0.59–0.92), while they have low correlation with the *k*-mer LASSO and the metafeature regression (range 0.04–0.12)—which in turn show mild-low correlation (range 0.12–0.49). Thus, both *k*-mer LASSO and metafeature regression stand distant from each other and the other methods. The PSS_cla_ dataset is explicitly constructed to measure class-specific concordance, with very similar resistant and susceptible instances. However, for this reason, the PSS_cla_ becomes by design a challenging dataset for classification because the reads derived from susceptible genes all align well with other resistant genes in the same AMR class. Thus, the performance of all algorithms will tend to flatten. Nonetheless, the metafeature approach exhibits the highest median accuracy. Overall—pooling both resistant and susceptible for each AMR class—the *k*-mer LASSO median rate of correct predictions is 44% (IQR, 35–48%), the metafeature ridge 46% (IQR, 33–48%), DeepARG 44% (IQR, 36–47%), AMRPlusPlus 2.0 45% (IQR, 36–50%), and Meta-MARC 44% (IQR, 36–47%).

### AMR-meta has lower false-positive rate on negative examples than other algorithms

As a sensitivity analysis, to study how the different algorithms behave with the negative samples in PSS_cla_, we sample the negative genomes based on their similarity with the positive ones, using increasing matching thresholds, i.e., 70–75%, >75–80%, >80–85%, and >85–90%. The hypothesis is that the false-positive rate correlates with the threshold; i.e., algorithms tend to mis-classify non-AMR reads/genes that share high similarity with AMR genes. Because AMR-meta is specifically trained on both negative and positive examples, the expectation is that the algorithm will pick fewer false-positive results than other methods. We thus assemble datasets for each AMR class and for each of the 4 ranges of similarity percentage, with a cap of 250 random genomes per class. As expected, the false-positive rate increases with similarity, and the metafeature model is the method with the lowest median false-positive rate (0.02), followed by DeepARG (0.06), Meta-MARC (0.2), *k*-mer LASSO (0.23), and AMRPlusPlus (0.3). The full results, stratified by class and threshold ranges, are provided in Supplementary Fig. S1.

### AMR-meta ensemble for functional genomics

The Soil and Pediatric datasets come from functional metagenomics experiments that by design guarantee the presence of antibiotic resistance in a sequence sample because the sample is cultured on antibiotic-laden medium. However, sequenced reads can also contain other or unknown genes, which cannot be quantified. We consider here the hit rate, i.e., the proportion of sequence reads classified as resistant. Caution: a higher hit rate can signify that a method finds more AMR genes but also that a method finds more false-positive genes. Given that AMR-meta is designed to decrease the false-positive rate, we expect it to be the most conservative. Yet, to empirically identify a trade-off between the approaches, in addition to running each single model, we also built an ensemble using voting with *k*-mer LASSO, the metafeature regression, and the individual models’ predictions as input features (requiring ≥2 concordant predictions for classifying resistance).

**Figure 5:**
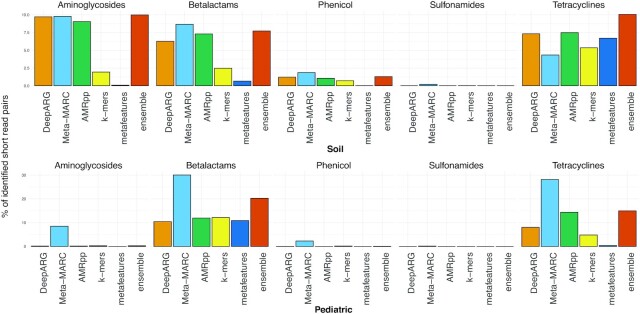
Percentage of sequence reads predicted resistant on the functional metagenomics data (Soil and Pediatric) by AMR-meta, off-the-shelf tools, and their voting ensemble.

On Soil, the voting ensemble achieves the highest hit rate, with a median fraction of read pairs identified as resistant of 7.72% (IQR, 1.28–10%), followed by AMRPlusPlus 2.0 with 7.03% (IQR, 1.06–7.48%), DeepARG with 6.27% (IQR, 1.21–7.32%), Meta-MARC with 4.97% (IQR, 1.86–8.68%), the *k*-mer approach with 1.94% (IQR, 0.7–2.49%), and the metafeature approach with 0.08% (IQR, 0.01–0.65%). On Pediatric, Meta-MARC achieves the highest hit rate with a median of 8.51% (IQR, 2.29–28.14%), followed by the *k*-mer approach with 0.27% (IQR, 0.2–4.8%), the voting ensemble with 0.27% (IQR, 0.05–4.97%), AMRPlusPlus 2.0 with 0.2% (IQR, 0.02–11.95%), DeepARG with 0.19% (IQR, 0.02–8.06%), and the metafeature approach with 0.01% (IQR, 0–0.4%). We observe large variations in each method depending on the class considered. It has to be noted that Meta-MARC’s threshold was previously re-calibrated on these datasets, and its standard threshold is much more conservative. As expected, the metafeature module is the most conservative on both datasets, while the voting ensemble offers a balanced alternative in all cases. Interestingly, the *k*-mer approach is one of the least conservative on the Pediatric set. Detailed results on the external Pediatric and Soil functional metagenomics datasets are illustrated in Fig. 5.

### Run-time comparison

To compare execution times, we create benchmark datasets of increasing size by selecting reads drawn from the semi-synthetic PATRIC data (across all classes), generating files of 1, 2, 5, and 10 GB of paired short-read files. We run all algorithms on the University of Florida’s High-Performance Cluster—HiPerGator 3.0—using a single node, composed by 4 AMD Opteron 6378 cores, with 32 GB of RAM. Table 2 shows run-times on the node. AMRPlusPlus 2.0 and AMR-meta *k*-mer LASSO are the fastest tools, with a time of execution difference within minutes up to 5 GB load. , and Meta-MARC is considerably slower (30-fold), hitting the 24-hour wall time for files >1 GB.

**Table 2:** Running times (hh:mm:ss) of AMR classification tools on metagenomics short-read data (reads drawn from the PATRIC datasets), 151 bases, paired end, fastq format

File size (R1+R2)	No. of reads (R1+R2)	AMR-meta (*k*-mer)	AMRPlusPlus 2.0	Meta-MARC	DeepARG
1GB	1,584,451	00:22:16	00:21:19	16:26:27	00:53:01
2 GB	3,168,014	00:43:37	00:49:40	>24 h	01:38:55
5 GB	7,924,402	01:47:24	01:35:47	>24 h	03:41:06
10 GB	15,851,366	03:32:46	02:48:43	>24 h	11:42:16

## Discussion

In this work, we present AMR-meta, an alignment-free, *k*-mer– and metafeature-based AMR classifier for short-read metagenomics data. AMR-meta uses an augmented training strategy based on non-AMR and AMR-homologous genes, providing relevant classification performance increment across various antibiotic classes.

Historically, the main objective of AMR characterization by metagenomics sequencing has been to identify known AMR genes, using comprehensive and up-to-date databases. However, the absence of non-AMR genes (negative examples) and of AMR-homologous sequences that do not have a role in resistance can hamper AMR classification accuracy and affect the trade-off between sensitivity and specificity. Notably, there are metagenomics classification tools that exploited the negative-positive *k*-mer representation paradigm. For instance, Clark weighs differently *k*-mers that are found only in specific species, as compared to those that are shared by different species or genera [42]. Other studies, focused on full-genome analysis and based on*in vitro* susceptibility, have shown high discriminating ability and capacity to identify potential new resistance features [27, 29].

It is worth mentioning that comparing different AMR tools can be challenging because not all use the same ontology or provide classifications at the same annotation level. For instance, Meta-MARC is trained on a self-determined similarity-based clustering of AMR genes, yet it is able to provide predictions at the mechanism/class/group level according to MEGARes ontology, matching the outputs of AMRPlusPlus 2.0 and AMR-meta. Instead, DeepARG uses a unique set of AMR categories derived from the CARD and ARDB. At this point, comparison of tools requires making an arbitrary choice on the AMR ontology to be used, and on the annotation level (e.g., class rather than mechanism), potentially penalizing one approach over another, as we show in our semi-synthetic PATRIC datasets PSS_mol_ and PSS_cla_. In addition, summarizing results over antibiotic classes can also introduce bias, given the high class imbalance in terms of antibiotics, gene frequency, and the aforementioned heterogeneity of intra-class gene diversity. It is understandable that a unified AMR ontology is difficult to achieve, yet an effort of the community to create common, standardized protocols for benchmarking and comparison is warranted.

One limitation of our approach is in the sample resistance/susceptibility annotation for validation and benchmark datasets. First, we label most of the bacterial genes that do not match to MEGARes as drug-susceptible, while in reality these sequences might contain new, undiscovered AMR genes. Second, there might be inconsistencies with antibiogram results in PATRIC.

Other limitations include the fact that we try only 1 metafeature approach—matrix factorization—while other methods could be tested, e.g., sparse binary principal/independent component analysis. Finally, it is known that *k*-mer approaches are not very sensitive to mutations, while mutant genes can still carry resistance.

Future development for AMR-meta including new strategies to select positive/negative labeled examples (and mutant genes) can further improve the classification performance. As another perspective, given the availability of efficient data structures for *k*-mer modeling, the LASSO module of AMR-meta could also be efficiently implemented as stand-alone AMR classifier to process data from portable sequencers in real time using mobile devices [43].

## Availability of Source Code and Requirements

- Project name: AMR-meta
- Project home page: https://github.com/smarini/AMR-meta
- RRID:SCR_022026
- biotoolsID: biotools:amr-meta
- Operating system: Linux
- Programming language: Bash, R, C++
- Other requirements: R packages Matrix, stringr, glmnet
- License: MIT

## Data Availability

As stated in the Methods, the datasets supporting the results of this article are obtainable from public sources, specifically Refseq, ncbi.nlm.nih.gov/refseq; MEGARes, megares.meglab.org; NCBI BioProject PRJNA244044, ncbi.nlm.nih.gov/bioproject/244044; NCBI BioProject PRJNA215106, ncbi.nlm.nih.gov/bioproject/215106; and PATRIC, patricbrc.org. The AMR-meta algorithm, including a containerized version via Singularity, is available at github.com/smarini/AMR-meta. Snapshots of our code and other data further supporting this work are openly available in the GigaScience respository, GigaDB [44].

## Additional Files

**Supplementary Figure S1**: Cumulative FP ratio over PSScla subsets. Negative short reads from the set genomes have been collated according to the growing similarity of BLAST matches, i.e., 70–75%, >75–80%, >80–85%, and >85–90%. FP ratio has been calculated for each subset. The figure reports the cumulative FP ratio for the different thresholds. Sets not represented in the figure did not provide genomes with similarities within the corresponding column ranges.

giac029_GIGA-D-21-00267_Original_Submission

giac029_GIGA-D-21-00267_Revision_1

giac029_GIGA-D-21-00267_Revision_2

giac029_Response_to_Reviewer_Comments_Original_Submission

giac029_Response_to_Reviewer_Comments_Revision_1

giac029_Reviewer_1_Report_Original_Submission Jacob Luber -- 10/13/2021 Reviewed

giac029_Reviewer_2_Report_Original_Submission Kamil Khanipov -- 10/24/2021 Reviewed

giac029_Supplemental_File

## Abbreviations

AMR: antimicrobial resistance; ARDB: Antibiotic Resistance Genes Database; BLAST: Basic Local Alignment Search Tool; BWA: Burrows-Wheeler Aligner; CARD: Comprehensive Antibiotic Resistance Database; DIAMOND: double index alignment of next-generation sequencing data; LASSO: least absolute shrinkage and selection operator; PATRIC: Pathosystems Resource Integration Center; IQR: interquartile range; HMM: hidden Markov model; UNIPROT: Universal Protein Resource.

## Competing Interests

The authors declare that they have no competing interests.

## Funding

This work was supported by the National Institutes of Health NIAID R01AI141810; the National Science Foundation SCH 2013998; and the United States Department of Agriculture AFRI 2019-67017-29110.

## Authors' Contributions

S.M., M.P., C.B., N.R.N., and T.K. conceived the idea and wrote the manuscript. S.M., M.O., I.B.S., and R.A.D. prepared the data and performed the experiments. All authors read and approved the final manuscript.
