# Supplementary material for: AMR-meta: a k-mer and metafeature approach to classify antimicrobial resistance from high-throughput short-read metagenomics data
Source: Gigascience. 2022 May 18;11:giac029. doi: 10.1093/gigascience/giac029 (PMC9116207; doi:10.1093/gigascience/giac029)
Supplement: giac029_GIGA-D-21-00267_Revision_1 [file giac029_giga-d-21-00267_revision_1.pdf]

GigaScience

AMR-meta: ak-mer and metafeature approach toclassify antimicrobial resistance fromhigh-throughput short-read metagenomics data

--Manuscript Draft--

**Manuscript Number:**	GIGA-D-21-00267R1
**Full Title:**	AMR-meta: ak-mer and metafeature approach toclassify antimicrobial resistance fromhigh-throughput short-read metagenomics data
**Article Type:**	Research
**Funding Information:**	National Institutes of Health (R01AI145552) — Dr. Mattia Prosperi<br>National Institutes of Health (R01AI141810) — Dr. Christina Boucher<br>National Science Foundation (SCH 201399) — Dr. Mattia Prosperi<br>U.S. Department of Agriculture (AFRI 2019-67017-29110) — Dr. Noelle Robertson Noyes
**Abstract:**	Background. Antimicrobial resistance (AMR) is a global health concern. High-throughput metagenomic sequencing of microbial samples enables profiling of AMR genes through comparison with curated AMR databases. However, performance of current methods are often hampered by database incompleteness, and presence of homology/homoplasy with other non-AMR genes in sequenced samples.<br>Results. We present AMR-meta, a database-free and alignment-free approach, based on k -mers, which combines algebraic matrix factorization into metafeatures with regularized regression. Metafeatures capture multi-level gene diversity across main antibiotic classes. AMR-meta takes in reads from metagenomic shotgun sequencing and outputs predictions about whether those reads contribute to resistance against specific classes of antibiotics. In addition, AMR-meta employs an augmented training strategy that joins an AMR gene database with non-AMR genes (used as negative examples). We compare AMR-meta with AMRPlusPlus, DeepARG, and Meta-MARC, further testing their ensemble via a voting system. In cross-validation, AMR-meta has a median (interquartile) f-score of 0.7 (0.2-0.9). On semi-synthetic metagenomic data --external test-- on average AMR-meta yields a 1.3-fold hit rate increase over existing methods. In terms of run-time, AMR-meta is 3x faster than DeepARG and 30x faster than Meta-MARC, and as fast as AMRPlusPlus. Finally, we note that differences in AMR ontologies and observed variance of all tools in classification outputs call for further development on standardization of benchmarking data and protocols.<br>Conclusions. AMR-meta is a fast, accurate classifier that exploits non-AMR negative sets to improve sensitivity and specificity. The differences in AMR ontologies and the high variance of all tools in classification outputs call for the deployment of standard benchmarking data and protocols, to fairly compare AMR prediction tools.
**Corresponding Author:**	Simone Marini<br>University of Florida<br>gainesville, FL UNITED STATES
**Corresponding Author Secondary Information:**	
**Corresponding Author's Institution:**	University of Florida
**Corresponding Author's Secondary Institution:**	
**First Author:**	Simone Marini
**First Author Secondary Information:**	
**Order of Authors:**	Simone Marini
	Marco Oliva
	Ilya Slizovskiy

	Rishabh Aryan Das
	Noelle Robertson Noyes
	Tamer Kahveci
	Christina Boucher
	Mattia Prosperi
**Order of Authors Secondary Information:**	
**Response to Reviewers:**	Please refer to our detailed response included in the main documents.
**Additional Information:**	
**Question**	**Response**
Are you submitting this manuscript to a special series or article collection?	Yes
Please select an option from the menu: as follow-up to "Are you submitting this manuscript to a special series or article collection?"	Functional Metagenomics
**Experimental design and statistics**<br><br>Full details of the experimental design and statistical methods used should be given in the Methods section, as detailed in our Minimum Standards Reporting Checklist. Information essential to interpreting the data presented should be made available in the figure legends.<br><br>Have you included all the information requested in your manuscript?	Yes
**Resources**<br><br>A description of all resources used, including antibodies, cell lines, animals and software tools, with enough information to allow them to be uniquely identified, should be included in the Methods section. Authors are strongly encouraged to cite Research Resource Identifiers (RRIDs) for antibodies, model organisms and tools, where possible.<br><br>Have you included the information requested as detailed in our Minimum Standards Reporting Checklist?	Yes

**Availability of data and materials**<br><br>All datasets and code on which the conclusions of the paper rely must be either included in your submission or deposited in publicly available repositories (where available and ethically appropriate), referencing such data using a unique identifier in the references and in the "Availability of Data and Materials" section of your manuscript.<br><br>Have you have met the above requirement as detailed in our Minimum Standards Reporting Checklist?	Yes

Powered by Editorial Manager® and ProduXion Manager® from Aries Systems Corporation

Manuscript
Click here to access/download;Manuscript;GigaScience_red_version.pdf

GigaScience, 0000, 1–11

doi: xx.xxxx/xxxx
Manuscript in Preparation
Paper

PAPER

AMR-meta: a *k*-mer and metafeature approach to classify antimicrobial resistance from high-throughput short-read metagenomics data

Simone Marini[1,*], Marco Oliva[2], Ilya B. Slizovskiy[3], Rishabh A. Das[1], Noelle Robertson Noyes[3], Tamer Kahveci[2], Christina Boucher[2] and Mattia Prosperi[1,*]

[1]Department of Epidemiology, University of Florida, and [2]Department of Computer and Information Science and Engineering, University of Florida, and [3]Department of Veterinary Population Medicine, University of Minnesota

* {simone.marini, m.prosperi}@ufl.edu

Abstract

Background Antimicrobial resistance (AMR) is a global health concern. High-throughput metagenomic sequencing of microbial samples enables profiling of AMR genes through comparison with curated AMR databases. However, performance of current methods are often hampered by database incompleteness, and presence of homology/homoplasy with other non-AMR genes in sequenced samples.
Results We present AMR-meta, a database-free and alignment-free approach, based on *k*-mers, which combines algebraic matrix factorization into metafeatures with regularized regression. Metafeatures capture multi-level gene diversity across main antibiotic classes. AMR-meta takes in reads from metagenomic shotgun sequencing and outputs predictions about whether those reads contribute to resistance against specific classes of antibiotics. In addition, AMR-meta employs an augmented training strategy that joins an AMR gene database with non-AMR genes (used as negative examples). We compare AMR-meta with AMRPlusPlus, DeepARG, and Meta-MARC, further testing their ensemble via a voting system. In cross-validation, AMR-meta has a median (interquartile) f-score of 0.7 (0.2-0.9). On semi-synthetic metagenomic data –external test– on average AMR-meta yields a 1.3-fold hit rate increase over existing methods. In terms of run-time, AMR-meta is 3x faster than DeepARG and 30x faster than Meta-MARC, and as fast as AMRPlusPlus. Finally, we note that differences in AMR ontologies and observed variance of all tools in classification outputs call for further development on standardization of benchmarking data and protocols.
Conclusions AMR-meta is a fast, accurate classifier that exploits non-AMR negative sets to improve sensitivity and specificity. The differences in AMR ontologies and the high variance of all tools in classification outputs call for the deployment of standard benchmarking data and protocols, to fairly compare AMR prediction tools.

Key words: functional metagenomics; short reads; antimicrobial resistance; machine learning; matrix factorization

Introduction

Antimicrobial resistance (AMR) is the ability of microorganisms to resist the effect of drugs targeted to eliminate them [1], and is globally recognized as a threat to public health as it makes treatment of microbial infections harder, increasing the risk of disease spread and severity [2]. Data from 890 U.S. hospitals collected on specific combinations of antibiotics and bac-

Compiled on: January 27, 2022.
Draft manuscript prepared by the author.

Key Points

- AMR-meta is a novel, database-free and alignment-free approach, combining matrix factorization with a training strategy including an AMR gene database plus non-AMR genes.
- On cross-validated results, AMR-meta has a median f-score of 0.7, while on external test sets it yields a 1.3-fold hit rate increase over existing methods.
- AMR-met is 3x to 30x faster than state-of-the art algorithms.

teria show that AMR caused an estimated 622,390 infections in 2017 [3]. Treating infections caused by AMR is clinically challenging since it requires to identify which drugs the infecting strain is susceptible to, and then to take a timely decision on the therapy to use. Notably, AMR is not limited to healthcare, as it represents a significant challenge also in animal and plant health, and thus in the entire ecosystem [4]. Therefore, detecting AMR in clinical, veterinarian, and botanical isolates is pivotal to curb the spread of AMR pathogens and reduce its impact. Although culture-based methods can accurately detect AMR, they are resource intensive with respect to trained personnel, monetary cost, and time [5]. Moreover, since only a fraction of bacterial species are cultivable with standard methods, culture-based methods are only applicable to a small number of bacteria. For these reasons, whole genome and metagenomics sequencing has become an increasingly prevalent method for AMR characterization. The challenge that then arises is how to accurately identify and quantify the AMR genes from such sequencing data. To accomplish this, a number of different methods have been proposed. Despite the concordance between in silico genotypic and in vitro phenotypic resistance assessment, the uptake of AMR prediction tools for routine healthcare has been slow, and they showed discordant performance in clinical settings [6].

AMR prediction methods for metagenomics rely on comparison to databases of AMR genes. Two comprehensive and widely used of AMR databases are the Comprehensive Antibiotic Resistance Database (CARD) [7, 8] and MEGARes [9, 10]. CARD is thoroughly maintained, with monthly updates on AMR determinants that have (i) an associated peer-reviewed scientific publication, (ii) a DNA sequence available in GenBank, (iii) clear experimental evidence of elevated minimum inhibitory concentration over controls. Currently, CARD integrates over 3,000 reference sequences of AMR genes and over 1,500 single nucleotide polymorphisms, knowledge on resistance mechanisms, and specific antibiotic classes. CARD uses a manually curated process and ontology, named the Antibiotic Resistance Ontology (ARO, github.com/arpcard/aro), which describes the molecular relations of antibiotic resistance (e.g., acquired resistance genes, drug targets, AMR mechanisms). MEGARes [9] -and its most recent 2.0 update [10]- is a hand-curated AMR database designed for high throughput sequencing data processing. MEGARes includes CARD genes and variants, but utilizes a different annotation structure. Specifically, it is a multi-level hierarchy (type, mechanism, class, group) in the form of a direct acyclic graph, ensuring that two higher level ranks are not linked to the same lower level rank. MEGARes annotation graph is therefore an optimal structure for ecological profiling and construction of AMR classifiers because, for example, it cannot result in conflicting sequence classification. MEGARes 2.0 currently includes ~8,000 genes. Major improvements from its first release consist in the inclusion of antibacterial biocide and metal resistance genes.

For AMR classification of metagenomic samples from high-throughput sequencing, one class of methods is based on the use of sequence read aligners. One widely used tool in this category is AMRPlusPlus [9], which aligns all reads to MEGARes using Burrows-Wheeler Aligner (BWA) [11] and then post-processes the alignment to identify the genes that have over 80% coverage from the alignment, providing the associated AMR annotation in the output. AMRPlusPlus 2.0 [10] is an improved version of AMRPlusPlus that is designed to be faster for large-scale projects. AMRPlusPlus 2.0 provides a post-alignment classification through the ResistomeAnalyzer (quality measure for nucleotide coverage of a reference sequence for a given read) and the RarefactionAnalysis (assessment of sequencing depth) modules. It also incorporates prediction of AMR due to single nucleotide polymorphisms in housekeeping genes, using a curated set that matches CARD. Of note, CARD also performs AMR prediction for housekeeping genes via the Resistant Gene Identifier (RGI), available as a web-service and a command-line application. Although alignment-based methods have high precision [12], they can only classify reads which align to known AMR genes. Given that existing AMR databases are incomplete, a large portion of novel AMR genes may go undetected.

Another class of methods for AMR characterization is alignment-free, employing a variety of approaches including substring (k-mer) matching and machine learning. ResFinder [13] and KmerResistance [14] process metagenomic reads by first constructing the set of all unique k-length subsequences (called k-mer spectrum) from the dataset. ResFinder 4.0 compares the set of unique k-mers to detect AMR genes and AMR-related chromosomal gene mutations based on an reference database built on a collection of chromosomal point mutations in bacterial pathogens [15], resistance genes from the Antibiotic Resistance Genes Database (ARDB) [16] and other literature sources [17]. The user is required to input a specific bacterial species for which the resistance is searched. Eight bacterial species are available. KmerResistance, as ResFinder, compares the set of unique k-mers to an ad hoc gene AMR reference database derived from literature [18, 19]. Specifically, KmerResistance uses exact co-occurring k-mer matching between a query sequence and the database, with a "winner takes all" strategy, i.e., multiple k-mer occurrences on different genes are resolved by selecting the one with highest frequency. Next, a quality measure of a whole AMR gene match is defined as a probability function of coverage (i.e., fraction of the genome covered by at least one k-mer) and depth (i.e., average number of times the k-mers in the match). Similar to alignment-based methods, ResFinder and KmerResistance are also bound to identifying genes that are found in a specified database, and therefore, have limited ability to detect putative AMR sequences. Another limitation of the k-mer based approaches is the low flexibility with respect to sequencing errors [14], possibly increasing false negative rates in sequence classification.

Other alignment-free methods use machine learning classifiers to identify putative and known AMR genes, such as Resfams [20] and Meta-MARC [12], both based on hierarchical hidden Markov models (HMMs). Resfams [20] preprocesses high-throughput sequences by assembling them and translating the resulting contigs into amino acid sequences. Meta-

MARC can predict AMR for an input sequence (either a short read or a longer assembled contig), according to the resistance class, group, and mechanism hierarchy defined in the MEGARes hierarchical data structure. Specifically, Meta-MARC is an ensemble of HMMs, each trained on a group of genes from MEGARes. A classification is performed by aggregating predictions from the lowest level of the MEGARes annotation hierarchy towards the highest level. Meta-MARC achieves better sensitivity, specificity, fraction of classified high-throughput sequence data, and number of AMR classes identified when compared to alignment matches and Resfams. However, the performance of Meta-MARC with short read data is worse than classifying assembled contigs.

DeepARG [21] is a hybrid machine learning and alignment-based approach that leverages convolutional deep learning networks. The alignment module first translates the input sequences to amino acids and using DIAMOND [22], and then aligns the translated sequences to a custom AMR database created by merging CARD, ARDB [16], and manually selected AMR sequences from the Universal Protein Resource (UNIPROT). The deep learning model then predicts the AMR class for all aligned reads. Since the machine learning step is subsequent to the alignment one, de facto DeepARG suffers from the limitations of alignment-based AMR prediction algorithms.

For completeness, it is worth mentioning AMR gene identification methods that are not specifically designed for high-throughput short read metagenomic data. These methods take as input one or a combination of: single genes, specific genome strains, genomic or proteomic variants, and/or protein primary, secondary, or tertiary structures. Similar to the methods described previously, these methods use alignment and/or machine learning paradigms [23, 24, 25, 26, 27, 28, 29, 30]. These algorithms bind the user into performing one or more supplementary pre-processing steps on metagenomics data, not included into the algorithm, such as sequence alignment or assembly, sequence translations into proteins, or protein structure prediction. Because of the required pre-processing, these methods defy the very advantages provided by the alignment-free design. For further reference, Hendriksen et al. [31] provide a comprehensive review.

While our work focuses on raw short-read AMR classification, we duly note that in the wider field of computational microbiomics, a variety of bioinformatics approaches exist and can be combined at different levels, from the characterization of species diversity in commensal and pathogenic host-ecological settings, to the identification of *novel* AMR genes or genetic elements relevant to AMR mechanisms and evolution. The de novo assembly methods can reconstruct complete AMR genes from short read data, locate them within core genomes or mobile elements, and assemble new genes that could be associated with phenotypic resistance; for example, the MegaHIT project [32] assembled the world's largest collection of gut microbiome genes with functional characterization. Also, the de novo assembly methods can be used to preprocess raw short read data for AMR classification [29]. Fast alignment methods can be used as well to quickly identify genetic signatures or point mutations responsible for AMR, e.g., in housekeeping genes, and map very large metagenomics samples to databases of interest, such as 16S rRNA gene collections [33].

In this paper, we develop *AMR-meta*, a novel, alignment-free, AMR classification approach for high-throughput metagenomic data, based on k-mers and matrix factorization of k-mers. The matrix factorization produces a number of 'metafeatures' able to capture multiple levels of gene diversity within broad AMR classes. Importantly, and differently from existing methods, AMR-meta uses an augmented training strategy that incorporates non-AMR genes as negative examples. We show that our approach is competitive with state-of-the-art tools (i.e., AMRPlusPlus 2.0, Meta-MARC, and DeepARG) in classification performance and execution speed. Notably, AMR-meta captures resistance mechanics complementary to those found by other tools, which instead are more correlated to each other.

Methods

AMR-meta is trained and tested first on an internal dataset that –differently from other approaches– includes both AMR (named resistant) and non-AMR genes (named susceptible). The AMR genes are taken from MEGARes 2.0 [10], while non-AMR genes are chosen from Genbank's RefSeq and include (a) bacterial genes that are highly dissimilar to AMR genes, and (b) AMR-homologous sequences, i.e. sequences highly similar to AMR genes, but not known to be associated to antibiotic resistance. By including the non-AMR and AMR-homologous sequences, we aim to decrease the false positive calls and to increase the true negative rates. This internal dataset is split into a 70/30 training/test ratio, and AMR-meta components (k-mers and k-mer-derived metafeatures) are trained and tested accordingly (all performance measures reported in this paper are relative to test sets). Second, we generate two semi-synthetic external datasets, drawing bacterial genomes from the Pathosystems Resource Integration Center (PATRIC) [34], and simulating short read data. We derive two PATRIC datasets that represent drug resistance/susceptibility relative to specific molecules or antibiotic classes, called PSS_{mol} and PSS_{cla}, respectively. This twofold design allows us to benchmark AMR-meta against other existing tools –AMRPlusPlus 2.0, Meta-MARC, and DeepARG– in a a flexible way, since their outputs levels vary among antibiotic classes and more specific mechanisms. We use PSS_{mol} to score the AMR predictions, and PSS_{cla} to estimate the concordance of AMR-meta class predictions with those of other methods. Finally, we combine AMR-meta with the other tools, and evaluate their predictions on two functional metagenomic datasets that were sampled a clinical and environmental setting. Our internal/external workflow is summarized in Figure 1.

Feature encoding and prediction models

AMR-meta k-mer LASSO module

The baseline models of AMR-meta are logistic regressors –one for each antibiotic class– that use raw k-mers as input. Each model utilizes the whole class-specific k-mer spectrum (derived from the collated positive/negative training datasets), where each feature is a binary value, representing the presence or absence of a particular k-mer in the dataset. Given the high-dimensionality of the k-mer spectrum, we use least absolute shrinkage and selection operator (LASSO) regularization to reduce the feature space, optimizing the shrinkage operator via cross-validation [35]. Given the heterogeneity in gene diversity within each class, e.g. Betalactamases have higher diversity than Floroquinolones, we also expect different cardinality of non-zero coefficients among the class-specific k-mer LASSO regressors.

AMR-meta metafeature ridge module

One possible problem with k-mer LASSO regression is that a single linear combination of k-mer features might not be able to explain the variance of the entire dataset, even if discrimination performance is good for the majority of genes in one class. A way to increase the portion of variance explained is to use more than one linear combination, e.g., the first m-th vectors of a principal component analysis. In this way, multiple independent combinations of k-mers can more effectively

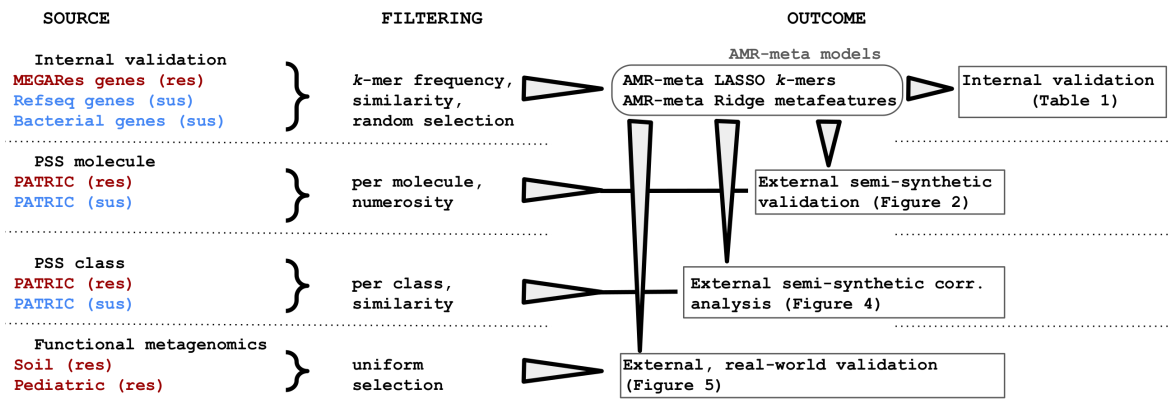

Figure 1. AMR-meta training/test workflow. We assemble an internal dataset of AMR and non-AMR homologous genes from MEGARes and RefSeq genes, on which AMR-meta models (k-mers, and metafeatures through matrix factorization) are trained and tested (70/30 split). AMR-meta and other AMR classification tools are then externally tested on: (i) semi-synthetic data from PATRIC at both antibiotic class and molecule levels (PSS_{cla} and PSS_{mol}); and (ii) functional metagenomics data (Soil and Pediatric).

represent the genetic diversity within antibiotic classes.

Accordingly, we explore a space transformation –with concomitant dimension reduction– of the k-mer spectrum that identifies a set of (orthogonal) multiple features, i.e., metafeatures, each as an independent combination of the original k-mers contributing to a cumulative portion of the data variance. To do so, we apply a matrix factorization approach, which has been previously shown apt to tackle complex feature extraction problems, e.g., oncology and proteomics [36, 37]. The method is based on non-negative matrix tri-factorization [38]. The algorithm identifies low-rank, non-negative matrices whose product provides an approximation of the original non-negative matrix.

Here we consider two data domains, namely k-mers and genes. A k-mer is related to a gene if it is present in the gene sequence. Let us denote the total number of genes with g; the total number of k-mers with t; a matrix of r rows and c columns having all values equal to zero with $\varnothing_{r,c}$; and a matrix with one gene per row, and one k-mer per column $R_{g,t}$ with, and $R^T_{g,t}$ as its transpose. We denote the transpose of a matrix A with superscript T as A^T in the rest of this paper. We express the relation between the two domains by a symmetrical, four-block matrix $R = \begin{pmatrix} \varnothing_{g,g} & R_{g,t} \\ R^T_{g,t} & \varnothing_{t,t} \end{pmatrix}$, where non-diagonal block matrices represent the relation (intersections) between k-mers and genes. Note that in this context, the relation between elements is defined by design: We set the value of a R at an entry to 1 if the corresponding k-mer is present in the corresponding gene, and 0 otherwise.

We denote the number of k-mer metafeatures and the number of gene metafeatures as m_t and m_g, respectively. The factorization procedure decomposes R into the product of three matrices G, S, and G^T, such that $G \times S \times G^T$ will approximate R by reducing the error up to a user-defined lower bound set as the difference between two consecutive iterations (denoted with $R \approx GSG^T$). Here G represents the relation between the original domains (genes, k-mers) and their metafeatures; and S represents the relation between the metafeatures, i.e., how one domain is mapped to the other. The matrices G and S have the following form both expressed as four block matrices:

$G = \begin{pmatrix} G_{g,m_g} & \varnothing_{g,m_t} \\ \varnothing_{k,m_g} & G_{t,m_t} \end{pmatrix}$ and $S = \begin{pmatrix} S_{m_g,m_g} & S_{m_g,m_t} \\ S_{m_t,m_g} & S_{m_t,m_t} \end{pmatrix}$.

We use the intersection between the data of the same domain as constraints in the factorization process, i.e., each domain has a block, symmetrical constraint. We define the matrix Θ to represent the self-domain relations, i.e., gene/gene and k-mer/k-mer relations. Therefore, Θ is an $R \times R$ matrix. The empty blocks of Θ are the non-diagonal blocks. $\Theta = \begin{pmatrix} \Theta_{g,g} & \varnothing_{g,t} \\ \varnothing_{t,g} & \Theta_{t,t} \end{pmatrix}$.

In Θ we set each entry to −1 if the corresponding row and column elements share a relation; 1 if unrelated; and 0 if the relation is unknown. In this application, in the Θ_t block we consider each k-mer identical to itself (related, −1), while we make no assumption about the relation with two different k-mers (not related, 0). In the Θ_g block, we consider all the genes of each class to be related (−1), and different from the genes of other classes (1).

The goal of the factorization is to minimize the following objective function:

$$min_{G \geq 0}(G; S) = \sum ||R_{ij} - G_i S_{ij} G^t_j|| + tr(G \Theta G^t) \quad (1)$$

where $||\cdot||$ indicates the Frobenius norm, and $tr(\cdot)$ indicates the trace. The objective function is composed of two parts: The first part measures the difference between the original matrix and the product of the three factorized matrices; the second part calculates the adherence of the factorized metafeatures to the constraints, in our case based on the AMR resistance class. The factorization process proceeds in an iterative fashion until convergence to a local minimum, with convergence heuristically defined by observing the value of the objective function and the corresponding reconstruction error below a user-defined threshold [38, 36, 37]. We fix a threshold of 10^{-2} as the difference between consecutive iterations, or reaching 5,000 iterations, as stop criteria. Previous works discuss the method in detail [36, 37]; a dedicated github repository contains code and user manual github.com/smarini/MaDDA. The factorization process, calculated over the full length training genes, produces G_{t,m_t}, which is the matrix relating the k-mers to their metafeatures. For each short read pair encoded as binary vector of k-mer occurrences $sr_{1,t}$, we calculate its metafeatures as $sr_{1,t} \times G_{t,m_t}$. Since the optimal number of metafeatures can be hard to infer, and the sizes of the matrices grow with the number of features [36, 37], for this application we used up to $m_t = 100$ and $m_g = 25$ metafeatures. After factorization, we feed the metafeatures to a logistic regression, optimizing the coefficients with a ridge approach. Figure 2 provides a graphical representation of the factorization process.

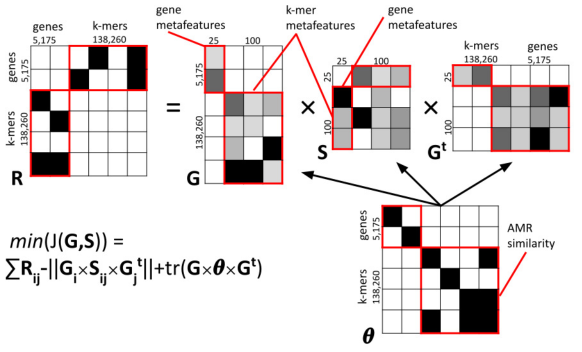

Figure 2. The matrix tri-factorization scheme. AMR, non-AMR, and AMR-homologous genes are paired up with k-mers across all antibiotic resistance classes into the R matrix, and the the dimension is reduced through the $R \approx GSG^t$ factorization, where the metafeatures are extracted, revealing the AMR similarity phenotypes in the θ matrix.

Training strategy

AMR genes.
We collate AMR genes from MEGARes 2.0 [10], constituting the positive (resistant) reference sets on the basis of the MEGARes annotation at the antibiotic class level. Of note, we exclude housekeeping genes that confer resistance through single point mutations.

Putative non-AMR bacterial genes.
We include putative non-AMR genes from the RefSeq database [39]. Using BLAST, we select the 1,000 RefSeq bacterial genes that do not match to MEGARes (e-value=10), aiming for a 1:1 target ratio with the antibiotic class of highest frequency. This gene set has high genetic divergence from the AMR genes in MEGARes, yet the nucleotide content is fully bacterial.

AMR-homologous human and vertebrate genes.
To mimic genes that likely do not provide AMR, but share a significant similarity with AMR genes we assemble a dataset selecting AMR-homologous genes and gene fragments from the human genome (GRCh38), and all the contigs in RefSeq labelled as 'vertebrate mammalian' and 'vertebrate other' assemblies. To do so, we run an ungapped BLAST search of all MEGARes genes against these human and vertebrate sequences (e-value=0.01). We use each unique sequence match, and add the flanking region to each match, elongating the matched sequence to be equal in length to the corresponding resistant MEGARes gene. Specifically, with a match of n_{match} nucleotides between target and query AMR gene, we extend the match by $\frac{n_{match}}{2}$ nucleotides in both directions on the target MEGARes sequence. The underlying assumption here is that matches of bacterial AMR genes on vertebrate genomes are spurious or not functional, and therefore do not provide AMR. Of note, this setup is similar to the test set derivation presented in DeepARG [21].

k-mer based and metafeature modelling.
All k-mers present in the genes of the training datasets, excluding any sample reserved for validation (see next subsection), are considered and counted using different values of k, from 13 to 77 based on prior literature evidence [14]. The best value for k is chosen incrementally on the basis of internal validation performance, stopping when performance decreases. Next, we stratify the training samples by class. We remove all k-mers with a frequency less than a given cut-off f in a single class (3 or 5 upon internal validation). We also exclude AMR classes with with less than 10 k-mers after frequency filtering.

Simulation of metagenomic short read data for training.
We use the AMR datasets described above to generate short reads, labelling each as resistant or susceptible to an antibiotic class. For each MEGARes class, we generate short read datasets providing 10x base coverage of the original full-gene data. These datasets allow the evaluation of both false positives and false negatives.

External validation

We use four independent external datasets, two semi-synthetic (made similarly to the training set), and two from functional metagenomic experiments. As the prevalence of AMR and the k-mer spectrum in the external test set is not guaranteed to be balanced as in the training, we re-calibrate the k-mer and metafeature probability threshold for external validation using the internal validation dataset and a number of samples where the k-mer and metafeature vectors are empty, i.e., they represent the non-AMR gene background. The ratio is optimized between 1:0.05 and 1:10, picking the first that meets the calibration target, i.e., a prediction with a score < 0.5 for a feature vector without any k-mer belonging to our model.

Semi-synthetic datasets.
We create the semi-synthetic datasets from PATRIC, downloading via FTP full bacterial genomes and summary metadata [30, 23]. We retain only genomes annotated as susceptible or resistant after an antibiogram test conform to the Clinical & Laboratory Standards Institute (CLSI), which is the most frequent testing standard in PATRIC, with over 55,000 resistant and 54,000 susceptible records [30]. Since the antibiotic nomenclature in PATRIC is molecule-specific and does not match exactly the MEGARes ontology hierarchy, we compile a lookup table linking each PATRIC drug annotation to a MEGARes class. We remove PATRIC genomes that do not refer to the AMR classes considered in the training phases, or are not included in the classes predicted by the concurrent methods.

We then generate two PATRIC semi-synthetic datasets (PSS), based on PATRIC antibiotic molecule labels (PSS_{mol}) and MEGARes classes (PSS_{cla}), respectively.

We use PSS_{mol} to assess the performance of our approach and the concurrent methods on molecule-specific data. We retain genomes are resistant (or susceptible) to at least one MEGARes class. We rank the PATRIC drug labels based on number of associated genomes, and we select the top ones based on the associated MEGARes classes. We exclude labels with less than 250 genomes, or labels not referring to a specific molecule (e.g., Tetracycline). We generate 250,000 short reads for each PATRIC label, equally divided between resistant and susceptible. Note that for PSS_{mol}, as the PATRIC labels refer to genome (and not the specific gene, as in MEGARes), it is not possible to determine the ground truth, i.e., if a short read comes from a resistant or a susceptible gene. To assess methods' performances, in absence of such ground truth, we develop a scoring system based on the assumption that a method should find more resistant read pairs from resistant genomes, and less from susceptible genomes. With $sr_{res,res}$ defined as the number of short read pairs coming from resistant genomes and classified as resistant, and with $sr_{res,sus}$ as the number of short read pairs coming from susceptible genomes and classified as resistant, we define the S-score as $S = sr_{res,res} - sr_{res,sus}$. A higher S-score thus denotes better performance, and a negative value implies that the method finds more resistant short read pairs among the susceptible ones.

PSS_{cla} is collated at the class level. Unlike PSS_{mol}, each short

read from PSS_{cla} has a known label which indicates if it comes from a resistant or susceptible gene. To generate PSS_{cla}, first we remove PATRIC genomes presenting inconsistent class annotations, i.e., that are annotated as both resistant and susceptible to antibiotics belonging to the same class. Second, in order to consider only genomes that are resistant (or susceptible) to the range of antibiotics within a given MEGARes class, we rank each genome in decreasing order of the total number of annotations of resistance (or susceptibility) to multiple drugs within the same class. Based on this ranking, we retain only genomes that rank over the 90th percentile. Third, we perform a class-by-class BLAST filtering (e-value=0.01, percent identity $\in [70, 90]$) of the selected PATRIC genomes against MEGARes genes, retaining and clipping the unique genes of PATRIC genomes that match MEGARes. The objective is to extract a set of PATRIC genes that match to MEGARes genes, but are not exact matches. In fact, genes similar to known resistant genes coming from antibiotic susceptible –by a phenotypic test– genomes represent excellent candidates to test the ability of classifier to recognize true/false positives. From these selected PATRIC genes, we generate short reads covering the selected genes, and capping the number of resistant or susceptible paired reads up to 100,000 per AMR class (i.e., 400,000 total reads per class). We reckon that with this procedure, we are able to label uniquely each PATRIC instance that passes the filter; however, in the BLAST alignment, there could be flanking regions or inserts that produce artifact matches. Nonetheless, given the strict parameters used, we we deem these cases to be rare. A resistant sample likely contains only resistant reads, and vice-versa for a susceptible sample. Therefore, it is possible to calculate sample-wide performance by counting the proportion of resistant-within-resistant and susceptible-within-susceptible reads in each test sample. After filtering, Glycopeptides and Lipopeptides are excluded as there are less than fifteen resistant genomes. Sulfonamides are excluded as no susceptible genomes is retained by our filtering procedure.

Functional metagenomics data.

We benchmarked our method against two functional metagenomic datasets, which we refer to as the Pediatric and the Soil datasets (NCBI BioProject Accessions PRJNA244044 and PRJNA215106). A functional metagenomics experiments is made by cloning metagenomic DNA fragments into bacterial vectors grown on antibiotic-laden media. The cultured bacteria surviving the antibiotic are sequenced using a clonally amplified high-throughput sequence library. As per experimental design, for each fosmid, all sequence reads contain at least one AMR gene (known or not yet discovered) resistant to a known antibiotic. Therefore, each sequencing experiment has a known antibiotic resistance label. However, since the original metagenomics fragments can be longer than a single AMR gene, a single fosmid might contain multiple AMR genes, or contain unknown genes. The Pediatric and Soil datasets include fosmids from Escherichia coli (DH10B) and consist of of 219 and 169 samples with an average of 1.98 and 1.12 million paired-end short reads respectively, sequenced with Illumina Genome Analyzer IIx technology. We utilize the aforementioned PATRIC annotation lookup table to pair antibiotic annotations to MEGARes classes. For testing classifiers' performance, we randomly select 100,000 short read pairs for each class as for the PATRIC datasets.

Software and hardware setup

We process the training/validation data, the semi-synthetic PSS_{mol} and PSS_{cla} datasets, and the experimental functional metagenomics data through in house UNIX scripts, off-the-shelf bioinformatics tools including BLAST, R (r-project.org/), and Bioconductor (bioconductor.org/). The k-mer LASSO and the metafeature regression are developed in R, bash, and C++. We download the functional metagenomics datasets using NCBI's sra-toolkit. For short read generation, we use InSilicoSeq [40], simulating Illumina's NovaSeq (company's top-line production scale sequencing instrument) reads with default parameters. We exclude genes shorter than 151 bases (length of NovaSeq's short reads) from the simulations. Code and R scripts are available publicly at github.com/smarini/AMR-meta under the MIT license.

Results

AMR-meta provides competitive prediction performance on multiple AMR classes

We generate thirteen datasets, corresponding to the antibiotic classes (according to the MEGARes ontology) of: Aminoglycosides, Betalactamases, Drug and biocide resistance, Fluoroquinolones, Glycopeptides, Lipopeptides, Macrolide-Lincosamide-Streptogramin (MLS), Multi-biocide resistance, Multi-drug resistance, Multi-metal-resistance, Phenicols, Sulfonamides, and Tetracyclines. We exclude classes with less than 10 k-mers after frequency filtering. Upon internal validation, the best k-mer length k and frequency threshold f are 13 and 5, respectively (the performance decreases at k=31 and for f=3 with the same or higher k). Upon optimization of the k value, the total number of unique 13-mers is 138,260, and the median (interquartile range, IQR) number per class is 3,645 (1,658-7,168). The matrix factorization includes 5,175 training genes, yielding a matrix R of 138,260 + 5,175 = 143,435 rows and columns, and a k-mer/metafeature matrix of $138,260 \times 100$ elements.

Table 1 shows the class-specific performance summaries by k-mer and metafeature regression on the internal validation sets. On the internal validation set, the k-mer LASSO and the metafeature regression exhibit a good tradeoff between sensitivity and specificity at both k values. The median (IQR) number of features selected by k-mer LASSO is 12,783 (12,304 and 13,179). As expected, the highest number of non-zero coefficients is found in the Betalactamase class, which is the class with higher diversity and number of resistant genes in MEGARes. The same holds for the highest number of metafeatures with positive coefficients (note that each metafeature is derived from the matrix factorization described above, incorporating several hundred thousands k-mer/gene elements). In terms of performance, for LASSO, the median (IQR) f-measure across all classes is 0.7 (0.2-0.9), while for the metafeature regression, the median f-measure is 0.4 (0.2-0.7). For both methods, the best performing classes are Betalactamases, and Fluoroquinolones, while the most problematic are MLS, and Multi-biocide, -drug, and -metal resistance. Despite the k-mer LASSO having a higher median f-measure, the metafeature regression performs better in the problematic MLS and Drug and biocide classes, shows better sensitivity in Glycopeptides, and better Specificity in Fluoroquinolones and Lipopetides. For reference comparison, the median (IQR) f-measure across classes is 0.5 (0.3-0.7) for DeepARG, and 0.9 (0.9-1.0) for Meta-MARC, based on the original papers' validation results. AMRPlusPlus 2.0 does not report per-class results on test sets.

Table 1. Performance of k-mer LASSO and metafeature ridge regression in predicting antibiotic class susceptibility/resistance on the internal test sets (30% of full dataset). Results show f-measure, Matthew's correlations coefficient (MCC), sensitivity and specificity; also, the number of non-zero k-mer LASSO and positive metafeature ridge coefficients are shown.

		**k-mer LASSO**					**Metafeature ridge**				
**Antibiotic Class**	N (test)	#feat.	F-measure	MCC	Sens.	Spec.	#metaf.	F-measure	MCC	Sens.	Spec.
Aminoglycosides	4,920	13,162	**0.85**	**0.84**	**0.79**	**0.99**	54	0.58	0.54	0.57	0.97
Betalactamases	36,052	19,483	**0.96**	**0.93**	**0.94**	**0.99**	74	0.89	0.79	0.83	0.96
Drug and biocide resistance	5,055	13,064	0.36	0.39	**0.93**	**0.76**	56	**0.39**	**0.93**	0.7	0.66
Fluoroquinolones	1,286	11,462	**0.98**	**0.98**	**0.96**	**1**	50	0.9	0.9	0.92	**1**
Glycopeptides	3,200	12,700	**0.8**	**0.8**	0.7	**1**	54	0.23	0.27	**0.84**	0.75
Lipopeptides	1,084	12,356	**0.85**	**0.85**	**0.76**	**1**	43	0.8	0.8	0.73	**1**
Macrolide-Lincosamide-Streptogramin	2,210	14,064	0.2	0.28	**0.93**	0.77	54	**0.3**	**0.29**	0.38	**0.97**
Multi-biocide resistance	1,412	12,304	**0.13**	**0.2**	**0.88**	**0.76**	51	0.1	0.16	0.78	0.73
Multi-drug resistance	1,387	12,280	**0.13**	**0.21**	**0.91**	**0.77**	48	0.11	0.18	0.83	0.74
Multi-metal resistance	2,407	13,179	**0.21**	**0.28**	**0.92**	**0.76**	62	0.18	0.25	0.9	0.73
Phenicols	922	11,115	**0.74**	**0.74**	**0.66**	**1**	51	0.44	0.44	0.53	0.99
Sulfonamides	531	12,783	**0.75**	**0.78**	0.6	1	54	**0.75**	0.77	1	0.6
Tetracyclines	4,208	14,286	0.86	0.85	0.8	1	43	0.67	0.65	0.67	0.98

AMR-meta generalizes robustly on external, semi-synthetic datasets

The PSS_{mol} dataset includes twelve molecule labels incorporated into antibiotic classes, namely: ciprofloxacin and levofloxacin (Fluoroquinolones); gentamicin and amikacin (Aminoglycosides); ceftriaxone and ampicillin (Betalactamases); chloramphenicol (Phenicols); sulfisoxazole (Sulfonamides); erythromycin and azithromycin (MLS); tigecycline (Tetracyclines); and vancomycin (Glycopeptides). Performance results in terms of S-score, which summarizes the correct resistance and susceptible hits (the higher the better), are shown in Figure 3. The median (IQR) S-score for the k-mer LASSO is 285.5 (123.5, 540), and for the metafeature regression is 322 (73, 470). Meta-MARC scores 250 (72, 359.5), DeepARG scores 144.5 (43, 345), and AMRPlusPlus 2.0 scores -29 (-377.5, 210). Overall, our metafeature approach shows both the highest performance and stability, exhibiting also a positive score in the levofloxacine molecule, whereas all the other methods produce a negative score. The k-mer LASSO component ranks second, followed by the other off-the-shelf tools.

AMR-meta predictions complement those of existing algorithms

Next, we measure the correlation between the predictions of the two AMR-meta modules and the ones from the other algorithms. As PSS_{mol} does not have a per-gene defined ground truth, we assemble PSS_{cla}. The PSS_{cla} dataset includes six out of the thirteen MEGARes classes, namely Aminoglycosides, Betalactamases, Fluoroquinolones, MLS, Phenicols, and Tetracyclines. PSS_{cla} has instances from both positive (resistant) and negative (susceptible) genes. When we look at the class-specific concordance for each pair of tools using the the Spearman's rank correlation (Figure 4), PSS_{cla} shows that the algorithms behave differently. Specifically, DeepARG, Meta-MARC, and AMRPlusPlus 2.0 are highly correlated in most of antibiotic classes (range 0.59-0.92), while they have low correlation with the k-mer LASSO and the metafeature regression (range 0.04-0.12) -which in turn show mild-low correlation (range 0.12-0.49). Thus, both k-mer LASSO and metafeature regression stand distant from each other and the other methods. The PSS_{cla} dataset is explicitly constructed to measure class-specific concordance, with very similar resistant and susceptible instances. However, for this reason, the PSS_{cla} becomes by design a challenging dataset for classification, because the reads derived from susceptible genes all well align with other resistant genes in the same AMR class. Thus, the performance

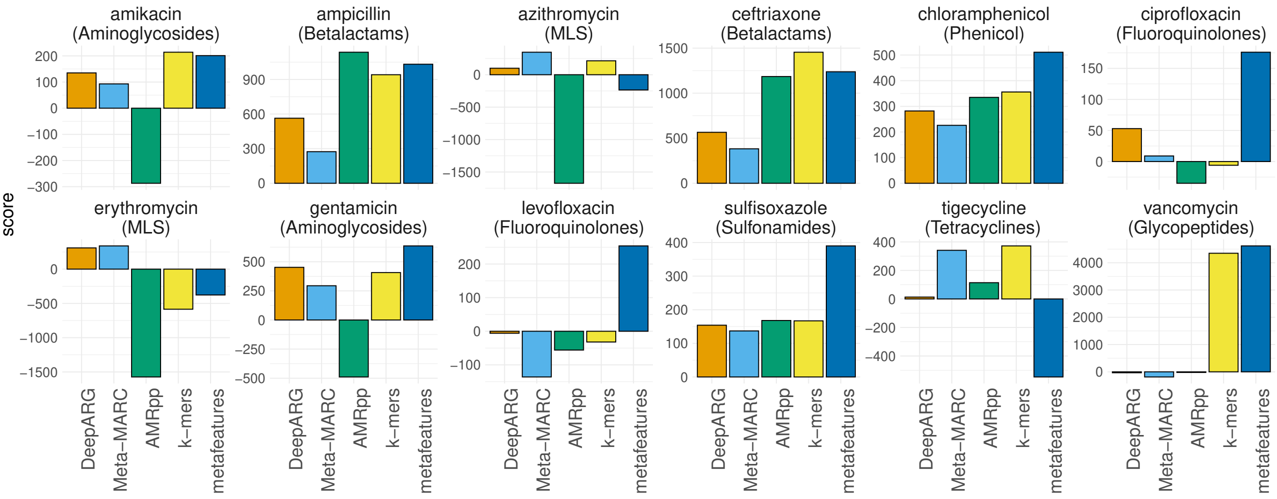

Figure 3. Performance of AMR-meta (k-mers and metafeatures) and of other off-the-shelf tools on the molecule-level PATRIC semi-synthetic data (PSS_{mol}). The S-score score is the difference between short read pairs predicted as resistant from the pooled resistant and susceptible genomes drawn from PATRIC.

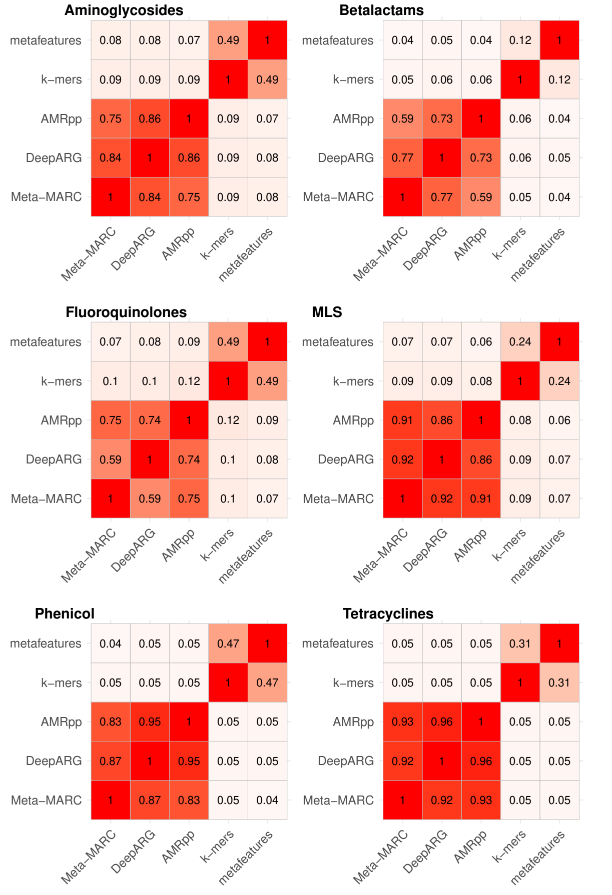

Figure 4. Spearman's Rank correlation of the AMR classifiers on the PATRIC semi-synthetic data (PSS_{cla}).

of all algorithms will tend to flatten. Nonetheless, the metafeature approach exhibits the highest median accuracy. Overall –pooling both resistant and susceptible for each AMR class– the *k*-mer LASSO median (IQR) percent of correct predictions is 44% (35%-48%), the metafeature ridge 46% (33%-48%), DeepARG 44% (36%-47%), AMRPlusPlus 2.0 45% (36%-50%), and Meta-MARC 44% (36%-47%).

AMR-meta has lower false positive rate on negative examples than other algorithms

As a sensitivity analysis, to study how the different algorithms behave with the negative samples in PSS_{cla}, we sample the negative genomes based on their similarity with the positive ones, using increasing matching thresholds, i.e., 70% to 75%, 75+% to 80%, 80+% to 85%, and 85+% to 90%. The hypothesis is that the false positive rate correlates with the threshold, i.e., algorithms tend to mis-classify non-AMR reads/genes that share high similarity with AMR genes. Since AMR-meta is specifically trained on both negative and positive examples, the expectation is that the algorithm will pick less false positives than other methods. We thus assemble datasets for each AMR class and for each of the four ranges of similarity percentage, with a cap of 250 random genomes per class. As expected, the false positive rate increases with similarity, and the metafeature model is the method with the lowest median false positive rate (0.02), followed by DeepARG (0.06), Meta-MARC (0.2), *k*-mer LASSO (0.23), and AMRPlusPlus (0.3). The full results, stratified by class and threshold ranges, are provided in Supplementary Figure S1.

AMR-meta ensemble for functional genomics

The Soil and Pediatric datasets come from functional metagenomics experiments that by design guarantee the presence of antibiotic resistance in a sequence sample, since the sample is cultured on antibiotic-laden medium. However, sequenced reads can also contain other or unknown genes, which cannot be quantified. We consider here the hit rate, i.e. the proportion of sequence reads classified as resistant. Cautionary, a higher hit rate can signify that a method finds more AMR genes, but also that a method finds more false positives. Given that AMR-meta is designed to decrease false positive rate, we expect it to be the most conservative. Yet, in order to identify empirically a tradeoff between the approaches, in addition to running each single model, we also built an ensemble using voting with *k*-mer LASSO, the metafeature regression, and the individual models' predictions as input features (requiring at least two concordant predictions for classifying resistance).

On Soil, the voting ensemble achieves the highest hit rate with a median (IQR) fraction of read pairs identified as resistant of 7.72% (1.28%-10%), followed by AMRPlusPlus 2.0 with 7.03% (1.06%-7.48%), DeepARG with 6.27% (1.21%-7.32%), Meta-MARC with 4.97% (1.86%-8.68%), the *k*-mer approach with 1.94% (0.7%-2.49%), and the metafeature approach with 0.08% (0.01%-0.65%). On Pediatric, Meta-MARC achieves the highest hit rate with a median (IQR) of 8.51% (2.29%-28.14%), followed by the *k*-mer approach with 0.27% (0.2%-4.8%), the voting ensemble with 0.27% (0.05%-4.97%), AMRPlusPlus 2.0 with 0.2% (0.02%-11.95%), DeepARG with 0.19% (0.02%-8.06%), and the metafeature approach with 0.01% (0%-0.4%). We observe large variations in each method depending on the class considered. It has to be noted that Meta-MARC's threshold was previously re-calibrated on these datasets, and its standard threshold is much more conservative. As expected, the metafeature module is the most conservative on both datasets, while the voting ensemble offers a balanced alternative in all cases. Interestingly, the *k*-mer approach is one of the least conservative on the Pediatric set. Detailed results on the external Pediatric and Soil functional metagenomics datasets are illustrated in Figure 5.

Run-time comparison

To compare execution times, we create benchmark datasets of increasing size by selecting reads drawn the semi-synthetic PATRIC data (across all classes), generating files of 1GB, 2GB, 5GB, and 10GB of paired short read files. We run all algorithms on University of Florida's High Performance Cluster – HiPerGator 3.0– using a single node, composed by four AMD Opteron 6378 cores, with 32GB of RAM. Table 2 show run times on the node. AMRPlusPlus 2.0 and MetAMR *k*-mer LASSO are the fastest tools, with a time of execution difference within minutes up to 5GB load. DeepARG is up to 3 times slower than MetAMR, and Meta-MARC is considerably slower (30-folds), hitting the 24-hour wall time for files larger than 1GB.

Discussion

In this work, we present AMR-meta, an alignment-free, *k*-mer- and metafeature-based AMR classifier for short read metagenomics data. AMR-meta uses an augmented training strategy based on non-AMR and AMR-homologous genes, providing relevant classification performance increment across various antibiotic classes.

Historically, the main objective of AMR characterization by metagenomics sequencing has been to identify known AMR

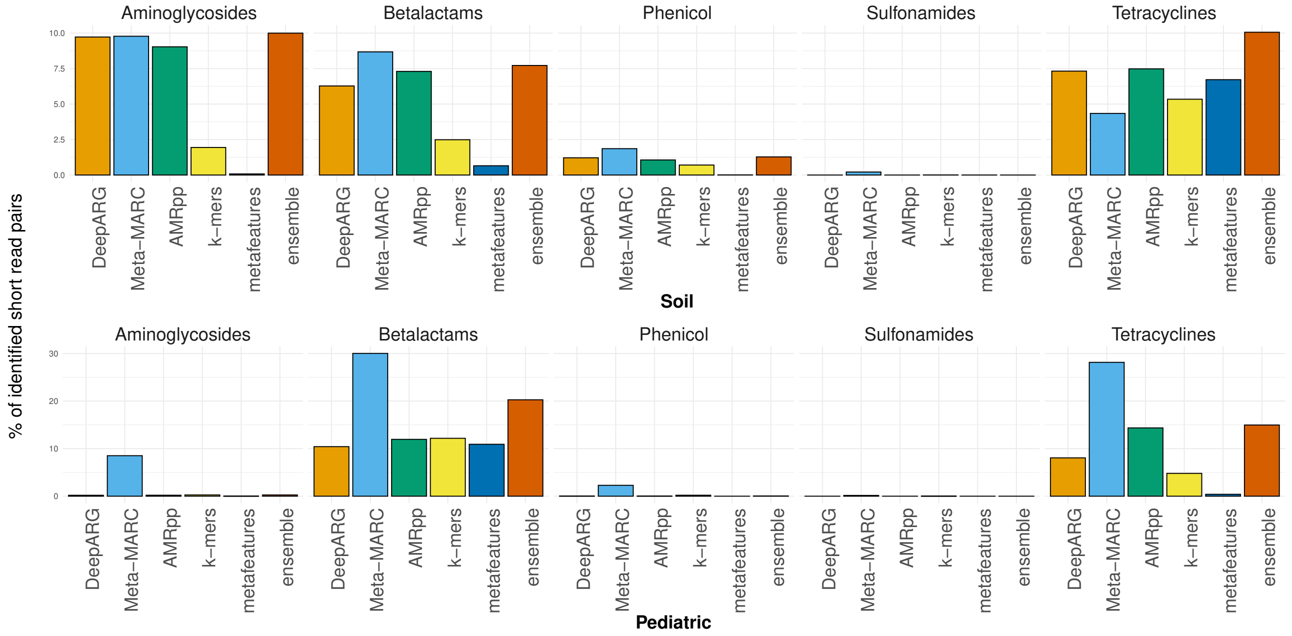

Figure 5. Percentage of sequence reads predicted resistant on the functional metagenomics data (Soil and Pediatric) by AMR-meta, off-the-shelf tools, and their voting ensemble.

Table 2. Running times (hh:mm:ss) of AMR classification tools on metagenomics short read data (reads drawn from the PATRIC datasets), 151 bases, paired end, fastq format).

File size (R1+R2)	# of reads (R1+R2)	AMR-meta (k-mer)	AMRPlusPlus 2.0	Meta-MARC	DeepARG
1GB	1,584,451	00:22:16	00:21:19	16:26:27	00:53:01
2GB	3,168,014	00:43:37	00:49:40	>24h	01:38:55
5GB	7,924,402	01:47:24	01:35:47	>24h	03:41:06
10GB	15,851,366	03:32:46	02:48:43	>24h	11:42:16

genes, using comprehensive and up-to-date databases. However, the absence of non-AMR genes (negative examples) and of AMR-homologous sequences that do not have role in resistance can hamper AMR classification accuracy, and impact the trade-off between sensitivity and specificity. Notably, there are metagenomics classification tools that exploited the negative-positive k-mer representation paradigm. For instance, Clark weighs differently k-mers that are found only in specific species, as compared to those that are shared by different species or genuses [41]. Other studies, focused on full-genome analysis and based on in vitro susceptibility, have shown high discriminating ability and capacity to identify potential new resistance features [27, 29].

It is worth to mention that comparing different AMR tools can be challenging, because not all use the same ontology, or provide classifications at the same annotation level. For instance, Meta-MARC is trained on a self-determined similarity-based clustering of AMR genes, yet it is able to provide predictions at the mechanism/class/group level according to MEGARes ontology, matching the outputs of AMRPlusPlus 2.0 and AMR-meta. Instead, DeepARG uses a unique set of AMR categories derived from the CARD and ARDB. At this point, comparison of tools requires making an arbitrary choice on the AMR ontology to be used, and on the annotation level (e.g. class rather than mechanism), potentially penalizing one approach over another, as we show in our semi-synthetic PATRIC datasets PSS_{mol} and PSS_{cla}. In addition, summarizing results over antibiotic classes can also introduce bias, given the high class imbalance in terms of antibiotics, gene frequency, and the aforementioned heterogeneity of intra-class gene diversity. It is understandable that a unified AMR ontology is difficult to achieve, yet an effort of the community to create common, standardized protocols for benchmarking and comparison is warranted.

One limitation of our approach is in the sample resistance/susceptibility annotation for validation and benchmark datasets. First, we label most of bacterial genes that do not match to MEGARes as drug-susceptible, whilst in reality these sequences might contain new, undiscovered AMR genes. Second, there might be inconsistencies with antibiogram results in PATRIC.

Other limitations include the fact that we try only one metafeature approach –matrix factorization– while other methods could be tested, e.g., sparse binary principal/independent component analysis. Finally, it is known that k-mer approaches are not very sensitive to mutations, while mutant genes can still carry resistance.

Future development for AMR-meta includes new strategies to select positive/negative labelled examples (and mutant genes) can further improve the classification performance. As another perspective, given the availability of efficient data structures for k-mer modelling, the LASSO module of AMR-meta could also be efficiently implemented as standalone AMR classifier to process data from portable sequencers in real time using mobile devices [42].

Availability of source code and requirements

- Project name: AMR-meta
- Project home page: https://github.com/smarini/AMR-meta
- Operating system(s): Linux
- Programming language: Bash, R, C++
- Other requirements: R packages Matrix, stringr, glmnet
- License: MIT

Availability of supporting data and materials

As stated in the Methods, the data sets supporting the results of this article are obtainable from public, specifically Refseq, ncbi.nlm.nih.gov/refseq; MEGARes, megares.meglab.org; NCBI BioProject PRJNA244044, ncbi.nlm.nih.gov/bioproject/244044; NCBI BioProject PRJNA215106 ncbi.nlm.nih.gov/bioproject/215106; and PATRIC, patricbrc.org. AMR-meta algorithm, including a containerized version via Singularity, is available at github.com/smarini/AMR-meta.

List of abbreviations

AMR - antimicrobial resistance ARDB - Antibiotic Resistance Genes Database BWA - Burrows-Wheeler Aligner CARD - Comprehensive Antibiotic Resistance Database DIAMOND - double index alignment of next-generation sequencing data LASSO - least absolute shrinkage and selection operator PATRIC - Pathosystems Resource Integration Center IQR - interquartile range HMM - hidden Markov model UNIPROT - Universal Protein Resource

Ethical Approval

Not applicable.

Consent for publication

Not applicable

Competing Interests

The authors declare that they have no competing interests.

Funding

This work was supported by the National Institutes of Health NIAID R01AI145552, NIAID R01AI141810; the National Science Foundation SCH 2013998; and the United States Department of Agriculture AFRI 2019-67017-29110.

Author's Contributions

SM, MP, CB, NRN, and TK conceived the idea and wrote the paper. SM, MO, IS, and RAD prepared the data and performed the experiments. All authors read and approved the final manuscript.

References

1. Von Wintersdorff CJ, Penders J, Van Niekerk JM, Mills ND, Majumder S, Van Alphen LB, et al. Dissemination of antimicrobial resistance in microbial ecosystems through horizontal gene transfer. Frontiers in Microbiology 2016;7:173.
2. Ventola CL. The antibiotic resistance crisis: part 1: causes and threats. Pharmacy and Therapeutics 2015;40(4):277.
3. Jernigan JA, Hatfield KM, Wolford H, Nelson RE, Olubajo B, Reddy SC, et al. Multidrug-resistant bacterial infections in US hospitalized patients, 2012–2017. New England Journal of Medicine 2020;382(14):1309–1319.
4. Nelson DW, Moore JE, Rao JR. Antimicrobial resistance (AMR): significance to food quality and safety. Food Quality and Safety 2019;3(1):15–22.
5. Hugenholtz P. Exploring prokaryotic diversity in the genomic era. Genome Biology 2002;3(2):reviews0003–1.
6. Doyle RM, O'Sullivan DM, Aller SD, Bruchmann S, Clark T, Pelegrin AC, et al. Discordant bioinformatic predictions of antimicrobial resistance from whole-genome sequencing data of bacterial isolates: An inter-laboratory study. Microbial Genomics 2020;6(2).
7. Jia B, Raphenya AR, Alcock B, Waglechner N, Guo P, Tsang KK, et al. CARD 2017: expansion and model-centric curation of the comprehensive antibiotic resistance database. Nucleic Acids Research 2016;p. gkw1004.
8. Alcock BP, Raphenya AR, Lau TT, Tsang KK, Bouchard M, Edalatmand A, et al. CARD 2020: antibiotic resistome surveillance with the comprehensive antibiotic resistance database. Nucleic Acids Research 2020;48(D1):D517–D525.
9. Lakin SM, Dean C, Noyes NR, Dettenwanger A, Ross AS, Doster E, et al. MEGARes: an antimicrobial resistance database for high throughput sequencing. Nucleic Acids Research 2017;45(D1):D574–D580.
10. Doster E, Lakin SM, Dean CJ, Wolfe C, Young JG, Boucher C, et al. MEGARes 2.0: a database for classification of antimicrobial drug, biocide and metal resistance determinants in metagenomic sequence data. Nucleic Acids Research 2020;48(D1):D561–D569.
11. Li H, Durbin R. Fast and accurate long-read alignment with Burrows–Wheeler transform. Bioinformatics 2010;26(5):589–595.
12. Lakin SM, Kuhnle A, Alipanahi B, Noyes NR, Dean C, Muggli M, et al. Hierarchical Hidden Markov models enable accurate and diverse detection of antimicrobial resistance sequences. Communications Biology 2019;2(1):1–11.
13. Bortolaia V, Kaas RS, Ruppe E, Roberts MC, Schwarz S, Cattoir V, et al. ResFinder 4.0 for predictions of phenotypes from genotypes. Journal of Antimicrobial Chemotherapy 2020;.
14. Clausen PT, Zankari E, Aarestrup FM, Lund O. Benchmarking of methods for identification of antimicrobial resistance genes in bacterial whole genome data. Journal of Antimicrobial Chemotherapy 2016;71(9):2484–2488.
15. Zankari E, Allesøe R, Joensen KG, Cavaco LM, Lund O, Aarestrup FM. PointFinder: a novel web tool for WGS-based detection of antimicrobial resistance associated with chromosomal point mutations in bacterial pathogens. Journal of Antimicrobial Chemotherapy 2017;72(10):2764–2768.
16. Liu B, Pop M. ARDB—antibiotic resistance genes database. Nucleic Acids Research 2009;37(suppl_1):D443–D447.
17. Zankari E, Hasman H, Cosentino S, Vestergaard M, Rasmussen S, Lund O, et al. Identification of acquired antimicrobial resistance genes. Journal of Antimicrobial Chemotherapy 2012;67(11):2640–2644.
18. Zankari E, Hasman H, Kaas RS, Seyfarth AM, Agersø Y, Lund O, et al. Genotyping using whole-genome sequencing is a realistic alternative to surveillance based on phenotypic antimicrobial susceptibility testing. Journal of Antimicrobial Chemotherapy 2013;68(4):771–777.
19. Stoesser N, Batty E, Eyre D, Morgan M, Wyllie D, Del Ojo Elias C, et al. Predicting antimicrobial susceptibilities

for Escherichia coli and Klebsiella pneumoniae isolates using whole genomic sequence data. Journal of Antimicrobial Chemotherapy 2013;68(10):2234–2244.

20. Gibson MK, Forsberg KJ, Dantas G. Improved annotation of antibiotic resistance determinants reveals microbial resistomes cluster by ecology. The ISME journal 2015;9(1):207–216.
21. Arango-Argoty G, Garner E, Pruden A, Heath LS, Vikesland P, Zhang L. DeepARG: a deep learning approach for predicting antibiotic resistance genes from metagenomic data. Microbiome 2018;6(1):1–15.
22. Buchfink B, Xie C, Huson DH. Fast and sensitive protein alignment using DIAMOND. Nature Methods 2015;12(1):59–60.
23. Davis JJ, Boisvert S, Brettin T, Kenyon RW, Mao C, Olson R, et al. Antimicrobial resistance prediction in PATRIC and RAST. Scientific Reports 2016;6:27930.
24. Kavvas ES, Catoiu E, Mih N, Yurkovich JT, Seif Y, Dillon N, et al. Machine learning and structural analysis of Mycobacterium tuberculosis pan-genome identifies genetic signatures of antibiotic resistance. Nature Communications 2018;9(1):1–9.
25. Srivastava A, Kumar R, Kumar M. BlaPred: Predicting and classifying β-lactamase using a 3-tier prediction system via Chou's general PseAAC. Journal of Theoretical Biology 2018;457:29–36.
26. Mahé P, El Azami M, Barlas P, Tournoud M. A large scale evaluation of TBProfiler and Mykrobe for antibiotic resistance prediction in Mycobacterium tuberculosis. PeerJ 2019;7:e6857.
27. Drouin A, Letarte G, Raymond F, Marchand M, Corbeil J, Laviolette F. Interpretable genotype-to-phenotype classifiers with performance guarantees. Scientific reports 2019;9(1):1–13.
28. Ruppé E, Ghozlane A, Tap J, Pons N, Alvarez AS, Maziers N, et al. Prediction of the intestinal resistome by a three-dimensional structure-based method. Nature Microbiology 2019;4(1):112–123.
29. Kim J, Greenberg DE, Pifer R, Jiang S, Xiao G, Shelburne SA, et al. VAMPr: VAriant Mapping and Prediction of antibiotic resistance via explainable features and machine learning. PLoS Computational Biology 2020;16(1):e1007511.
30. Marini S, Oliva M, Slizovskiy IB, Noyes NR, Boucher C, Prosperi M. Exploring Prediction of Antimicrobial Resistance Based on Protein Solvent Accessibility Variation. Frontiers in Genetics 2021;12:26.
31. Hendriksen RS, Bortolaia V, Tate H, Tyson G, Aarestrup FM, McDermott P. Using genomics to track global antimicrobial resistance. Frontiers in Public Health 2019;7:242.
32. Qin J, Li R, Raes J, Arumugam M, Burgdorf KS, Manichanh C, et al. A human gut microbial gene catalogue established by metagenomic sequencing. nature 2010;464(7285):59–65.
33. Consortium HMP, et al. Structure, function and diversity of the healthy human microbiome. nature 2012;486(7402):207.
34. Davis JJ, Wattam AR, Aziz RK, Brettin T, Butler R, Butler RM, et al. The PATRIC Bioinformatics Resource Center: expanding data and analysis capabilities. Nucleic Acids Research 2020;48(D1):D606–D612.
35. Simon N, Friedman J, Hastie T, Tibshirani R. Regularization Paths for Cox's Proportional Hazards Model via Coordinate Descent. Journal of Statistical Software 2011;39(5):1–13.
36. Vitali F, Marini S, Pala D, Demartini A, Montoli S, Zambelli A, et al. Patient similarity by joint matrix trifactorization to identify subgroups in acute myeloid leukemia. JAMIA Open 2018;1(1):75–86.
37. Marini S, Vitali F, Rampazzi S, Demartini A, Akutsu T. Protease target prediction via matrix factorization. Bioinformatics 2019;35(6):923–929.
38. Žitnik M, Zupan B. Data fusion by matrix factorization. IEEE Transactions on Pattern Analysis and Machine Intelligence 2014;37(1):41–53.
39. O'Leary NA, Wright MW, Brister JR, Ciufo S, Haddad D, McVeigh R, et al. Reference sequence (RefSeq) database at NCBI: current status, taxonomic expansion, and functional annotation. Nucleic Acids Research 2016;44(D1):D733–D745.
40. Gourlé H, Karlsson-Lindsjö O, Hayer J, Bongcam-Rudloff E. Simulating Illumina metagenomic data with InSilicoSeq. Bioinformatics 2019;35(3):521–522.
41. Ounit R, Wanamaker S, Close TJ, Lonardi S. CLARK: fast and accurate classification of metagenomic and genomic sequences using discriminative k-mers. BMC Genomics 2015;16(1):236.
42. Oliva M, Milicchio F, King K, Benson G, Boucher C, Prosperi M. Portable Nanopore Analytics: Are We There Yet? Bioinformatics 2020;.

Click here to access/download
Supplementary Material
AMR-meta_Supplementary_Material.pdf

Personal Cover Click here to access/download;Personal Cover;GigaScience_rebuttal.pdf

We thank the Editor and the Reviewers for their comprehensive comments and suggestions, and appreciated the positive feedback. We revised the paper accordingly. We also apologize for the delay in resubmission, even if only minor revisions were called (our lab is a CoV sequencing hub and we had to redirect most resources to that).

Please find below the in-line answers to reviewers.

Reviewer #1

The beginning of the "Results" section of the abstract could benefit from the addition of one sentence that introduces what is being done for the benefit of readers who are not super familiar with the AMR subfield of computational metagenomics. "We present AMR-meta, a database-free and alignment-free approach, based on k-mers, which combines algebraic matrix factorization into metafeatures with regularized regression. Metafeatures capture multi-level gene diversity across main antibiotic classes." could be supplemented with a sentence that explains explicitly what the input and output of the algorithm is: "AMR-meta takes in reads from metagenomic shotgun sequencing and outputs predictions about whether those reads contribute to resistance against specific classes of antibiotics". By making the paper accessible at the entry, many people will cite the superb literature review section!

Re. Thanks. We revised the introductory paragraph and added the suggested sentence.

2) The literature review is comprehensive and superb. I would suggest adding two to three sentences comparing the development of competing ideologies (alignment vs de novo assembly) in the wider computational microbiome field to contextualize that these different philosophies apply in the context of AMR but also to all of ubiome research. Citing a seminal MetaHIT consortium paper and a seminal early Human Microbiome Project consortium paper to make this point would suffice. The literature review as it stands is incredibly impressive.

Re. We thank the reviewer for this comment. We added the following sentences to the literature review, citing the MetaHIT consortium the Human Microbiome Project consortium:

While our work focuses on raw short-read AMR classification, we duly note that in the wider field of computational microbiomics, a variety of bioinformatics approaches exist and can be combined at different levels, from the characterization of species diversity in commensal and pathogenic host-ecological settings, to the identification of *novel* AMR genes or genetic elements relevant to AMR mechanisms and evolution. The de novo assembly methods can reconstruct complete AMR genes from short read data, locate them within core genomes or mobile elements, and assemble new genes that could be associated with phenotypic resistance; for example, the MegaHIT project [32] assembled the world's largest collection of gut microbiome genes with functional characterization. Also, the de novo assembly methods can be used to preprocess raw short read data for AMR classification [29]. Fast alignment methods can be used as well to quickly identify genetic signatures or point mutations responsible for AMR,

e.g., in housekeeping genes, and map very large metagenomics samples to databases of interest, such as 16S rRNA gene collections [33].

The only additional analysis that I think the paper requires is to show that the design elements that are contributing to the models superb performance are: a) including both resistance and susceptible classes in training and b) the generation of metafeatures. To do this, I suggest:
i) Run the model with only the AMR negative genes as a baseline.
ii) conclusively show that metafeatures from having two classes (resistance and susceptible) are contributing to the model performance by doing a series of permutation tests, where each permutation test is generating many "AMR positive" sets sampled from pools of genes defined at a distinct percent identity (degree of homology) cut-offs (i.e. 95%, 90%, 70%, 50%) and show that the distribution of model performance results more closely starts to resemble the model from i) only trained on the AMR negative genes as the homology cutoffs for defining the AMR positive genes are loosened (by utilizing CD-HIT I can't imaging this analysis taking more than a day or so).

Re. We thank the reviewer for this interesting point. Following the reviewer's suggestion, we subset the negative genomes from the PATRIC dataset PSS_{cla} (for which we had phenotypic AMR as ground truth), and then sampled matching ones based on the similarity thresholds as indicated, with a cap of 250 random genomes per class. As explained in the methods, negative samples in this dataset show a BLAST similarity in the [70, 90] range. We assembled sets based on four ranges, i.e., similarity >75, 75+ to 80, 80+ to 85, and >85. As expected, the false positive rate correlated with the threshold. The metafeature model was the ones with the lowest median false positive rate (0.02), followed by DeepARG (0.06), Meta-Marc (0.2), k-mer (0.23), and AMRPlusPlus (0.3). We summarized this sensitivity analysis in the main text, and placed the detailed per-class and per-threshold results in the supplement, as follows:

As a sensitivity analysis, to study how the different algorithms behave with the negative samples in PSS_{cla}, we sample the negative genomes based on their similarity with the positive ones, using increasing matching thresholds, i.e., 70% to 75%, 75+% to 80%, 80+% to 85%, and 85+% to 90%. The hypothesis is that the false positive rate correlates with the threshold, i.e., algorithms tend to mis-classify non-AMR reads/genes that share high similarity with AMR genes. Since, AMR-meta is specifically trained on both negative and positive examples, the expectation is that the algorithm will pick less false positives than other methods. We thus assemble datasets for each AMR class and for each of the four ranges of similarity percentage, with a cap of 250 random genomes per class. As expected, the false positive rate increases with similarity, and the metafeature model is the method with the lowest median false positive rate (0.02), followed by DeepARG (0.06), Meta-MARC (0.2), k-mer LASSO (0.23), and AMRPlusPlus (0.3). The full results, stratified by class and threshold ranges, are provided in Supplementary Figure S1.

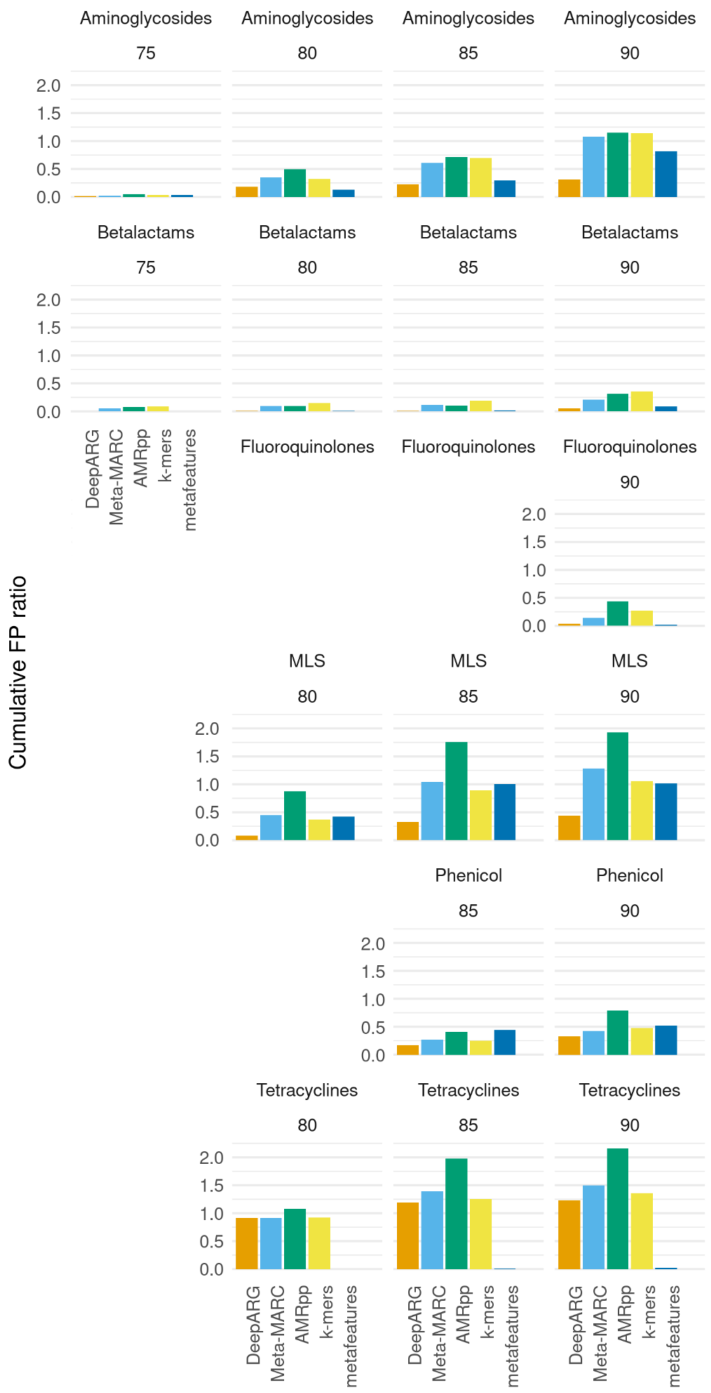

Supplementary Figure S1. Cumulative FP ratio over PSS_{cla} subsets. Negative short reads from the set genomes have been collated according to the growing similarity percentage of BLAST matches, i.e., 70% to 75%, 75+% to 80%, 80+% to 85%, and 85+% to 90%. FP ratio has been calculated for each subset. The figure reports the cumulative FP ratio for the different thresholds. Sets not represented in the figure did not provide genomes with similarities within the corresponding column ranges.

Reviewer #2

The authors present AMR-meta, a database-free and alignment-free approach, based on k-mers, which combines algebraic matrix factorization into metafeatures with regularized regression. AMR-meta is a fast, accurate classifier that exploits non-AMR negative sets to improve sensitivity and specificity. It offers same or significantly faster run time in comparison to other similar functioning tools. The tools was evaluated and compared to other available tools using synthetic and real world datasets. The manuscript compares the proposed development with other existing tools, highlighting the advantages and disadvantages of each one. The manuscript is written well and clearly.

Re. We thank the reviewer for the positive feedback.
