# Supplementary material for: AMR-meta: a k-mer and metafeature approach to classify antimicrobial resistance from high-throughput short-read metagenomics data
Source: Gigascience. 2022 May 18;11:giac029. doi: 10.1093/gigascience/giac029 (PMC9116207; doi:10.1093/gigascience/giac029)
Supplement: giac029_Supplemental_File [file giac029_supplemental_file.pdf]

Supplementary Material

AMR-meta: a k-mer and metafeature approach to classify antimicrobial resistance from high-throughput short-read metagenomics data

Simone Marini, Marco Oliva, Ilya B. Slizovskiy, Rishabh A. Das, Noelle Robertson Noyes, Tamer Kahveci, Christina Boucher and *Mattia Prosperi.*

Supplementary Figure S1 (next page). Cumulative FP ratio over PSS_{cla} subsets. Negative short reads from the set genomes have been collated according to the growing similarity of BLAST matches, i.e., 70% to 75%, 75+% to 80%, 80+% to 85%, and 85+% to 90%. FP ratio has been calculated for each subset. The figure reports the cumulative FP ratio for the different thresholds. Sets not represented in the figure did not provide genomes with similarities within the corresponding column ranges.

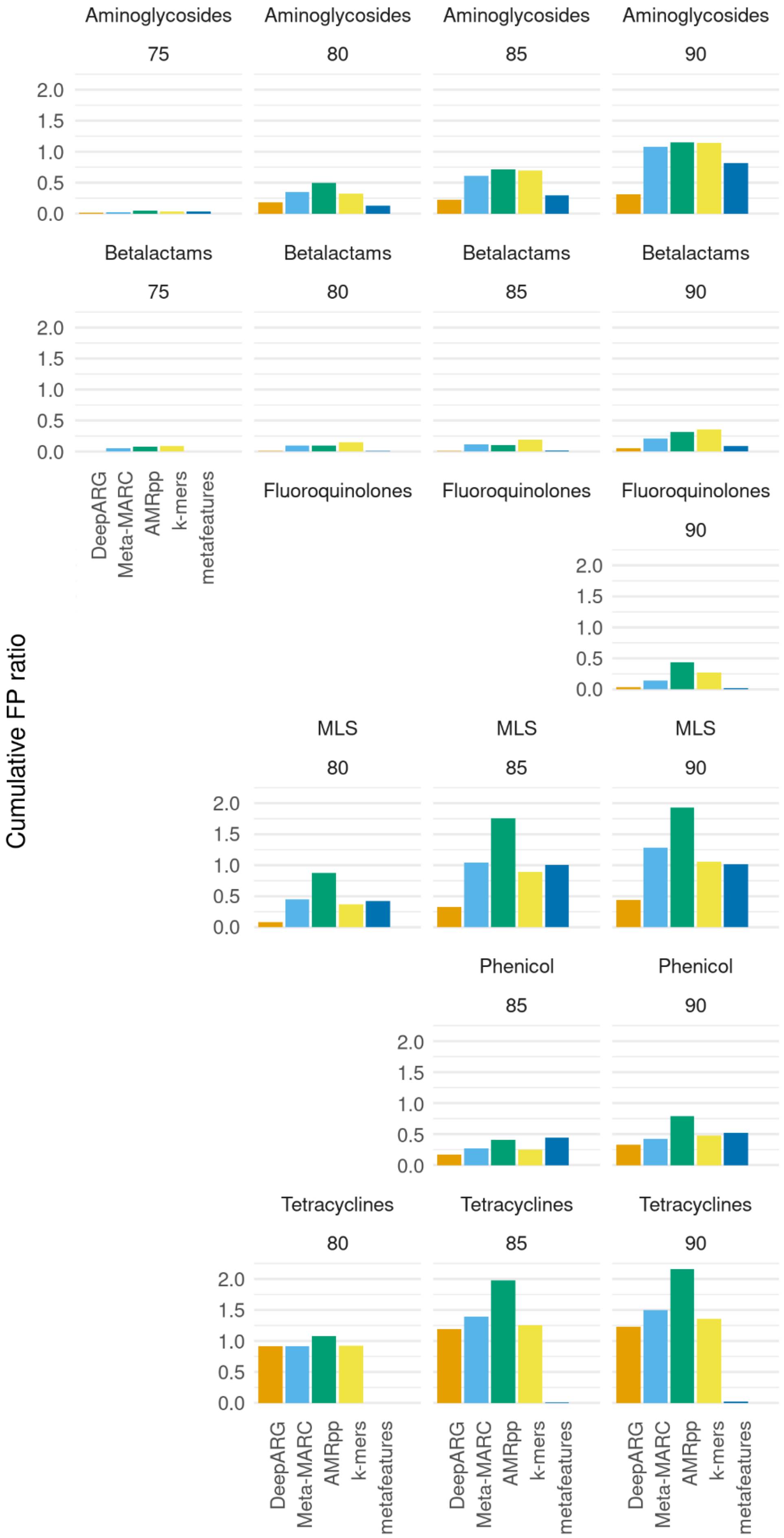

Aminoglycosides 75
Aminoglycosides 80
Aminoglycosides 85
Aminoglycosides 90
Betalactams 75
Betalactams 80
Betalactams 85
Betalactams 90
Fluoroquinolones
Fluoroquinolones
Fluoroquinolones 90
MLS 80
MLS 85
MLS 90
Phenicol 85
Phenicol 90
Tetracyclines 80
Tetracyclines 85
Tetracyclines 90
Cumulative FP ratio
0.0
0.5
1.0
1.5
2.0
DeepARG
Meta-MARC
AMRpp
k-mers
metafeatures
