# Supplementary material for: AMR-meta: a k-mer and metafeature approach to classify antimicrobial resistance from high-throughput short-read metagenomics data
Source: Gigascience. 2022 May 18;11:giac029. doi: 10.1093/gigascience/giac029 (PMC9116207; doi:10.1093/gigascience/giac029)
Supplement: giac029_Response_to_Reviewer_Comments_Original_Submission [file giac029_response_to_reviewer_comments_original_submission.pdf]

Author's Response To Reviewer Comments

Close

Please refer to our detailed response included in the main documents.

Close
