# Supplementary material for: AMR-meta: a k-mer and metafeature approach to classify antimicrobial resistance from high-throughput short-read metagenomics data
Source: Gigascience. 2022 May 18;11:giac029. doi: 10.1093/gigascience/giac029 (PMC9116207; doi:10.1093/gigascience/giac029)
Supplement: giac029_Response_to_Reviewer_Comments_Revision_1 [file giac029_response_to_reviewer_comments_revision_1.pdf]

Author's Response To Reviewer Comments

Close

We followed the formatting instruction as per Editorial communication on Feb 20, and:

- added The GigaDB DOI citation

- added the a new reference 43

- registered the software in the bio.tools and SciCrunch.org, indicating the RRID (Research Resource Identification Initiative ID) and biotoolsID identifiers

All changes in the main text are in red.

Also, please note that upon final revision, we removed one of our mentioned funding sources, as it was inserted by mistake. More specifically, we removed the mention to NIAID R01AI145552. This is a grant our team obtained, but it is related to HIV (not antimicrobial resistance).

Close
