# Supplementary material for: AMR-meta: a k-mer and metafeature approach to classify antimicrobial resistance from high-throughput short-read metagenomics data
Source: Gigascience. 2022 May 18;11:giac029. doi: 10.1093/gigascience/giac029 (PMC9116207; doi:10.1093/gigascience/giac029)
Supplement: giac029_Reviewer_1_Report_Original_Submission — Jacob Luber -- 10/13/2021 Reviewed [file giac029_reviewer_1_report_original_submission.pdf]

Reviewer Report

Title: AMR-meta: a k-mer and metafeature approach toclassify antimicrobial resistance fromhigh-throughput short-read metagenomics data

Version: Original Submission **Date:** 10/13/2021

Reviewer name: Jacob Luber

Reviewer Comments to Author:

This paper presents a database and alignment free approach based on k-mers to build a predictor based on metafeatures derived from matrix factorization to predict whether reads contribute to resistance against certain types of antibiotics. The paper presents novel computational methodologies that are refreshing in a sea of stale OTU and alignment based methods in the field of computational metagenomics. Specifically, the Tri-factorization scheme that the authors introduce generates metafeatures that allow for state of the art predictive performance. This paper will also provide tremendous value to readers because of how incredibly thorough the literature review portion is. I have 2 minor formatting suggestions and 1 minor additional analysis that I think would be useful for this excellent paper.

Minor Formatting Suggestions:

1) The beginning of the "Results" section of the abstract could benefit from the addition of one sentence that introduces what is being done for the benefit of readers who are not super familiar with the AMR subfield of computational metagenomics. "We present AMR-meta, a database-free and alignment-free approach, based on k-mers, which combines algebraic matrix factorization into metafeatures with regularized regression. Metafeatures capture multi-level gene diversity across main antibiotic classes." could be supplemented with a sentence that explains explicitly what the input and output of the algorithm is: "AMR-meta takes in reads from metagenomic shotgun sequencing and outputs predictions about whether those reads contribute to resistance against specific classes of antibiotics". By making the paper accessible at the entry, many people will cite the superb literature review section!

2) The literature review is comprehensive and superb. I would suggest adding two to three sentences comparing the development of competing ideologies (alignment vs de novo assembly) in the wider computational microbiome field to contextualize that these different philosophies apply in the context of AMR but also to all of ubiome research. Citing a seminal MetaHIT consortium paper and a seminal early Human Microbiome Project consortium paper to make this point would suffice. The literature review as it stands is incredibly impressive.

Minor Computational Analysis:

1) The only additional analysis that I think the paper requires is to show that the design elements that are contributing to the models superb performance are: a) including both resistance and susceptible classes in training and b) the generation of metafeatures. To do this, I suggest:

i) Run the model with only the AMR negative genes as a baseline.

ii) conclusively show that metafeatures from having two classes (resistance and susceptible) are contributing to the model performance by doing a series of permutation tests, where each permutation

test is generating many "AMR positive" sets sampled from pools of genes defined at a distinct percent identity (degree of homology) cut-offs (i.e. 95%, 90%, 70%, 50%) and show that the distribution of model performance results more closely starts to resemble the model from i) only trained on the AMR negative genes as the homology cutoffs for defining the AMR positive genes are loosened (by utilizing CD-HIT I can't imaging this analysis taking more than a day or so).
This is overall a great paper and I look forward to seeing it published!
Jacob M. Luber, Ph.D.
Assistant Professor of Computer Science, The University of Texas at Arlington
Assistant Professor of Bioengineering, The University of Texas at Arlington
Guest Researcher, NIH/NCI

Methods

Are the methods appropriate to the aims of the study, are they well described, and are necessary controls included? Choose an item.

Conclusions

Are the conclusions adequately supported by the data shown? Choose an item.

Reporting Standards

Does the manuscript adhere to the journal's guidelines on minimum standards of reporting? Choose an item.

Choose an item.

Statistics

Are you able to assess all statistics in the manuscript, including the appropriateness of statistical tests used? Choose an item.

Quality of Written English

Please indicate the quality of language in the manuscript: Choose an item.

Declaration of Competing Interests

Please complete a declaration of competing interests, considering the following questions:

- Have you in the past five years received reimbursements, fees, funding, or salary from an organisation that may in any way gain or lose financially from the publication of this manuscript, either now or in the future?
- Do you hold any stocks or shares in an organisation that may in any way gain or lose financially from the publication of this manuscript, either now or in the future?

- Do you hold or are you currently applying for any patents relating to the content of the manuscript?
- Have you received reimbursements, fees, funding, or salary from an organization that holds or has applied for patents relating to the content of the manuscript?
- Do you have any other financial competing interests?
- Do you have any non-financial competing interests in relation to this paper?

If you can answer no to all of the above, write 'I declare that I have no competing interests' below. If your reply is yes to any, please give details below.

I have a pending US patent that includes methods for using de novo assembly based approaches to predict host modulatory functionality in microbes (https://patentimages.storage.googleapis.com/f7/d2/f3/a4174373bd2bbc/WO2020172604A1.pdf).

I agree to the open peer review policy of the journal. I understand that my name will be included on my report to the authors and, if the manuscript is accepted for publication, my named report including any attachments I upload will be posted on the website along with the authors' responses. I agree for my report to be made available under an Open Access Creative Commons CC-BY license (http://creativecommons.org/licenses/by/4.0/). I understand that any comments which I do not wish to be included in my named report can be included as confidential comments to the editors, which will not be published.

Choose an item.

To further support our reviewers, we have joined with Publons, where you can gain additional credit to further highlight your hard work (see: https://publons.com/journal/530/gigascience). On publication of this paper, your review will be automatically added to Publons, you can then choose whether or not to claim your Publons credit. I understand this statement.

Yes Choose an item.
